# Supplementary material for: Osirisynes G-I, New Long-Chain Highly Oxygenated Polyacetylenes from the Mayotte Marine Sponge Haliclona sp
Source: Mar Drugs. 2020 Jul 3;18(7):350. doi: 10.3390/md18070350 (PMC7401255; doi:10.3390/md18070350)

Supporting information

Osirisynes G-I, New Long-chain Highly Oxygenated Polyacetylenes from the Mayotte Marine Sponge *Haliclona* sp.

Pierre-Eric Campos[1], Gaëtan Herbette[2], Christophe Chendo[2], Patricia Clerc[1], Florent Tintillier[1], Nicole J. de Voogd[3,4], Eleni-Dimitra Papanagnou[5], Ioannis P. Trougakos[5], Moran Jerabek[6], Jérôme Bignon[7], Géraldine Le Goff[7], Jamal Ouazzani[7] and Anne Gauvin-Bialecki[1]*

[1] Laboratoire de chimie et de biotechnologie des produits naturels, Faculté des Sciences et Technologies, Université de La Réunion, 15 Avenue René Cassin, CS 92003, 97744 Saint-Denis Cedex 9, La Réunion, France

[2] Aix-Marseille Univ, CNRS, Centrale Marseille, FSCM, Spectropole, Campus de St Jérôme-Service 511, 13397 Marseille, France

[3] Naturalis Biodiversity Center, Darwinweg 2, 2333 CR Leiden, Netherlands

[4] Institute of Environmental Sciences, Leiden University, Einsteinweg 2, 2333 CC Leiden, Netherlands

[5] Department of Cell Biology and Biophysics, Faculty of Biology, National and Kapodistrian University of Athens, Athens, 15784, Greece

[6] Crelux GmbH, Am Klopferspitz 19a, 82152 Planegg-Martinsried, Germany

[7] Institut de Chimie des Substances Naturelles, CNRS UPR 2301, Univ. Paris-Sud, Université Paris-Saclay, 1, av. de la Terrasse, 91198 Gif-sur-Yvette, France

Figure S1: HRESIMS spectrum for osirisyne G (**1**) 4
Figure S2: ^{1}H NMR (600 MHz, CD_3OD) spectrum for osirisyne G (**1**)........ 5
Figure S3: ^{1}H-^{1}H COSY NMR (600 MHz) spectrum for osirisyne G (**1**) 6
Figure S4: ^{1}H-^{13}C HSQC NMR (600 MHz) spectrum for osirisyne G (**1**) 7
Figure S5: ^{1}H-^{13}C HMBC NMR (600 MHz) spectrum for osirisyne G (**1**) 8
Figure S6: HRESIMS spectrum for osirisyne H (**2**) 9
Figure S7: ESI$^+$-MS/MS (S7.a.) and ESI$^-$-MS/MS (S7.b.) spectra of osirisyne H (**2**) with outlines of dissociation of the precursor ion........ 10
Figure S8*:* Dissociation mechanisms of the fragmentation of osirisyne H (**2**) in ESI$^+$-MS/MS with the mass *m/z* of the different fragments. 12
Figure S9: ^{1}H NMR (600 MHz, CD_3OD) spectrum for osirisyne H (**2**)........ 14
Figure S10: ^{1}H-^{1}H COSY NMR (600 MHz) spectrum for osirisyne H (**2**) 15
Figure S11: ^{1}H-^{13}C HSQC NMR (600 MHz) spectrum for osirisyne H (**2**) 16
Figure S12: ^{1}H-^{13}C HMBC NMR (600 MHz) spectrum for osirisyne H (**2**) 17
Figure S13: ^{1}H-^{13}C HSQC-TOCSY NMR (600 MHz) spectrum for osirisyne H (**2**) 18
Figure S14: HRESIMS spectrum for osirisyne I (**3**)........ 19
Figure S15*:* ESI$^+$-MS/MS (S13.a.) and ESI$^-$-MS/MS (S13.b.) spectra of osirisyne I (**3**) with outlines of dissociation of the precursor ion........ 20
Figure S16*:* Dissociation mechanisms of the fragmentation of osirisyne I (**3**) in ESI$^+$-MS/MS (14.a.) and ESI$^-$-MS/MS (14.b.) with the mass *m/z* of the different fragments........ 22
Figure S17: IRTF spectrum for osirisyne I (**3**)........ 25
Figure S18: ^{1}H NMR (600 MHz, CD_3OD) spectrum for osirisyne I (**3**) 26
Figure S19: ^{13}C NMR (600 MHz, CD_3OD) spectrum for osirisyne I (**3**) 27
Figure S20: ^{1}H-^{1}H COSY NMR (600 MHz) spectrum for osirisyne I (**3**) 28
Figure S21: ^{1}H-^{13}C HSQC NMR (600 MHz) spectrum for osirisyne I (**3**)........ 29
Figure S22: ^{1}H-^{13}C HMBC NMR (600 MHz) spectrum for osirisyne I (**3**)........ 30
Figure S23: ^{1}H-^{1}H NOESY NMR (600 MHz) spectrum for osirisyne I (**3**) 31
Figure S24: ^{1}H-^{13}C TOCSY-HSQC NMR (600 MHz) spectrum for osirisyne I (**3**) 32
Figure S25: HRESIMS spectrum for osirisyne A (**4**)........ 33
Figure S26*:* ESI$^+$-MS/MS (S22.a.) and ESI$^-$-MS/MS (S22.b.) spectra of osirisyne A (**4**) with outlines of dissociation of the precursor ion........ 34
Figure S27: ^{1}H NMR (600 MHz, CD_3OD) spectrum for osirisyne A (**4**)........ 36
Figure S28: ^{13}C NMR (600 MHz, CD_3OD) spectrum for osirisyne A (**4**)........ 37
Figure S29: HRESIMS spectrum for osirisyne B (**4**)........ 38
Figure S30*:* ESI$^+$-MS/MS (S25.a.) and ESI$^-$-MS/MS (S25.b.) spectra of osirisyne B (**5**) with outlines of dissociation of the precursor ion........ 39
Figure S31: ^{1}H NMR (600 MHz, CD_3OD) spectrum for osirisyne B (**5**)........ 41
Figure S32: ^{13}C NMR (600 MHz, CD_3OD) spectrum for osirisyne B (**5**)........ 42
Figure S33: ^{1}H-^{1}H TOCSY NMR (600 MHz) spectrum for osirisyne B (**5**) 43
Figure S34: HRESIMS spectrum for osirisyne E (**6**)........ 44
Figure S35*:* ESI$^+$-MS/MS (S28.a.) and ESI$^-$-MS/MS (S28.b.) spectra of osirisyne E (**6**) with outlines of dissociation of the precursor ion........ 45
Figure S36: ^{1}H NMR (600 MHz, CD_3OD) spectrum for osirisyne E (**6**) 47
Figure S37: ^{13}C NMR (600 MHz, CD_3OD) spectrum for osirisyne E (**6**) 48

In situ Haliclona sp. photo

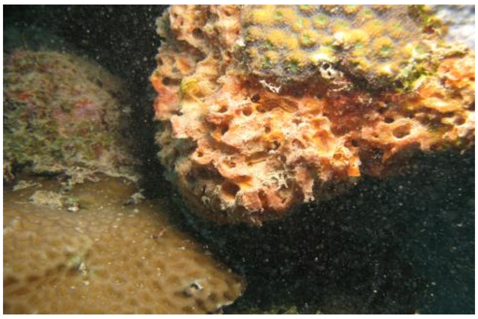

S0. Observed data for known compounds.

Osirisyne A (**4**): amorphous solid, ^{1}H and ^{13}C NMR data see **Supporting Information**; HRESIMS *m/z* 811.4996 $[M - H]^-$ (calcd for $C_{47}H_{71}O_{11}^-$, 811.5002).

Osirisyne B (**5**): amorphous solid, ^{1}H and ^{13}C NMR data see **Supporting Information**; HRESIMS *m/z* 795.5049 $[M - H]^-$ (calcd for $C_{47}H_{71}O_{10}^-$, 795.5053).

Osirisyne E (**6**): amorphous solid, ^{1}H and ^{13}C data see **Supporting Information**; HRESIMS *m/z* 795.5059 $[M - H]^-$ (calcd for $C_{47}H_{71}O_{10}^-$, 795.5053).

Figure S1: HRESIMS spectrum for osirisyne G (**1**)

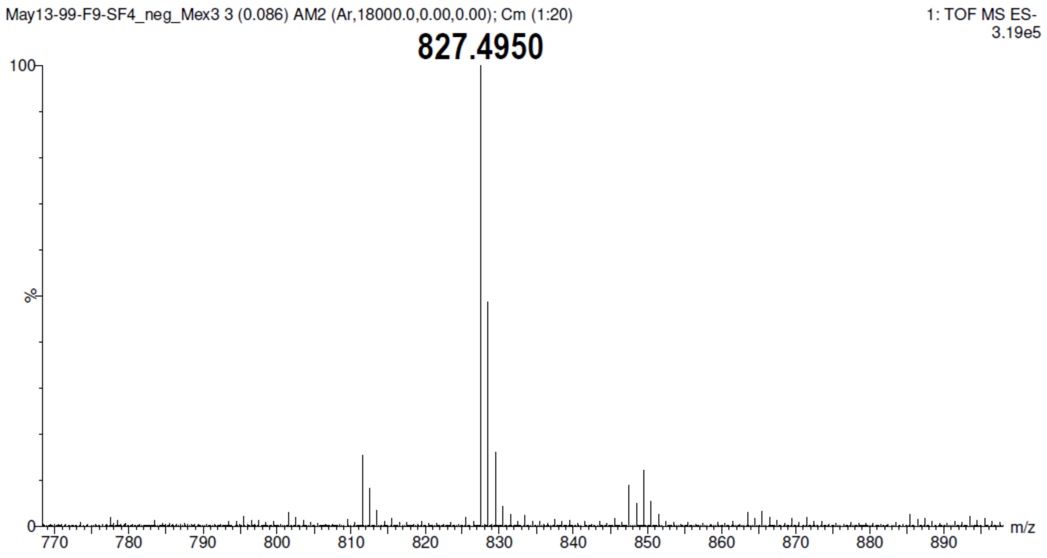

Figure S2: ^{1}H NMR (600 MHz, CD_3OD) spectrum for osirisyne G (**1**)

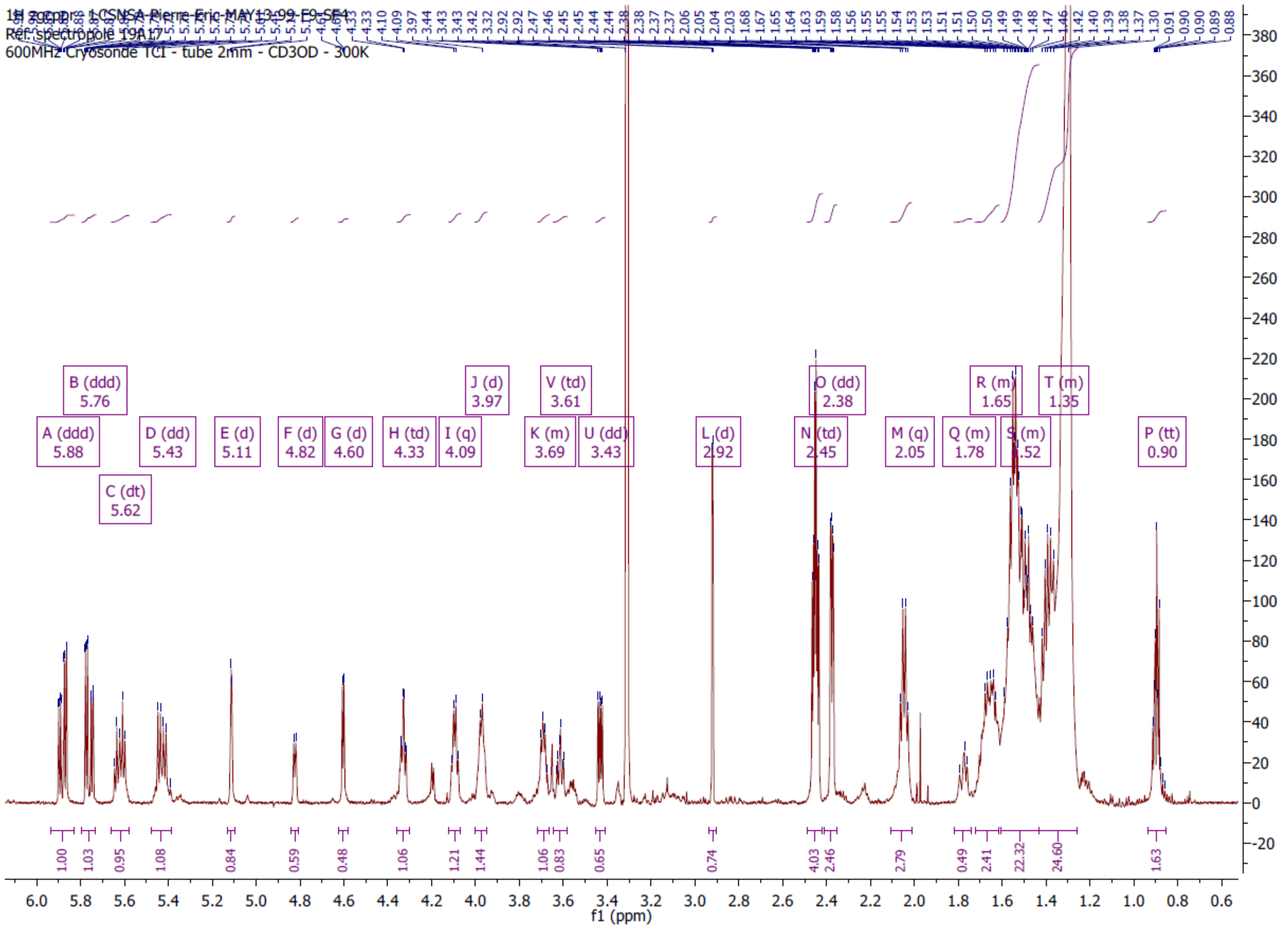

Figure S3: ^{1}H-^{1}H COSY NMR (600 MHz) spectrum for osirisyne G (**1**)

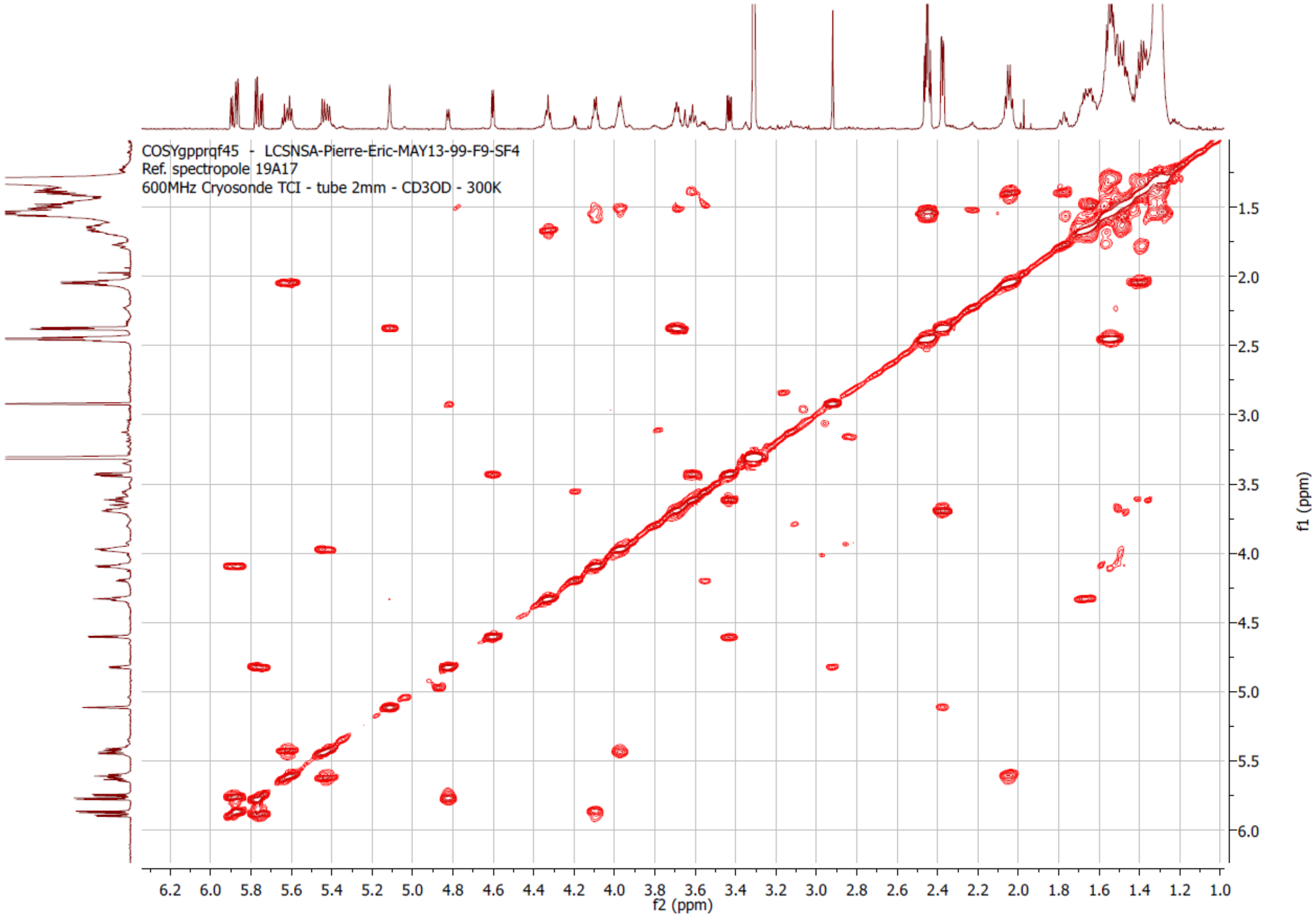

Figure S4: ^{1}H-^{13}C HSQC NMR (600 MHz) spectrum for osirisyne G (**1**)

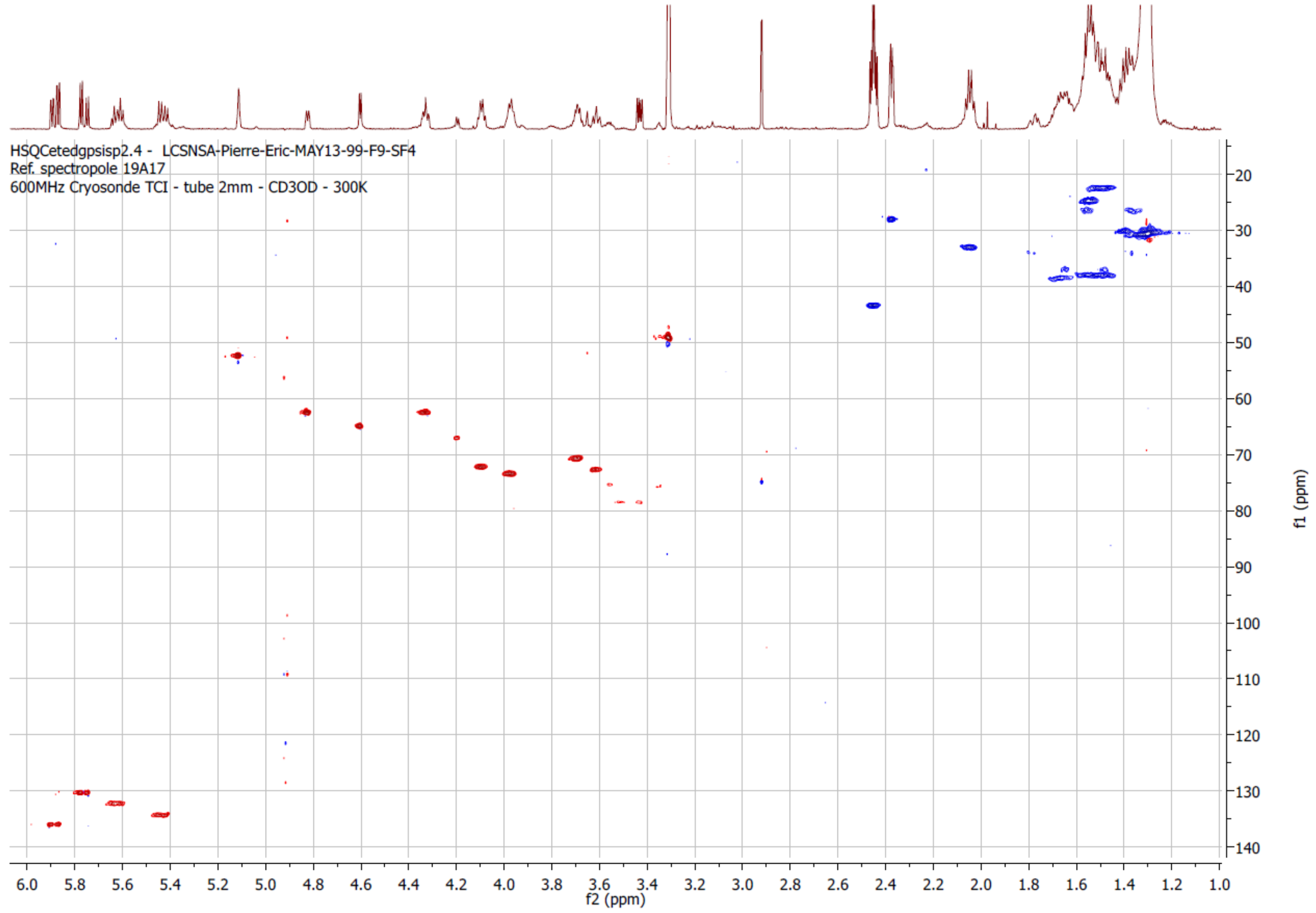

Figure S5: ^{1}H-^{13}C HMBC NMR (600 MHz) spectrum for osirisyne G (**1**)

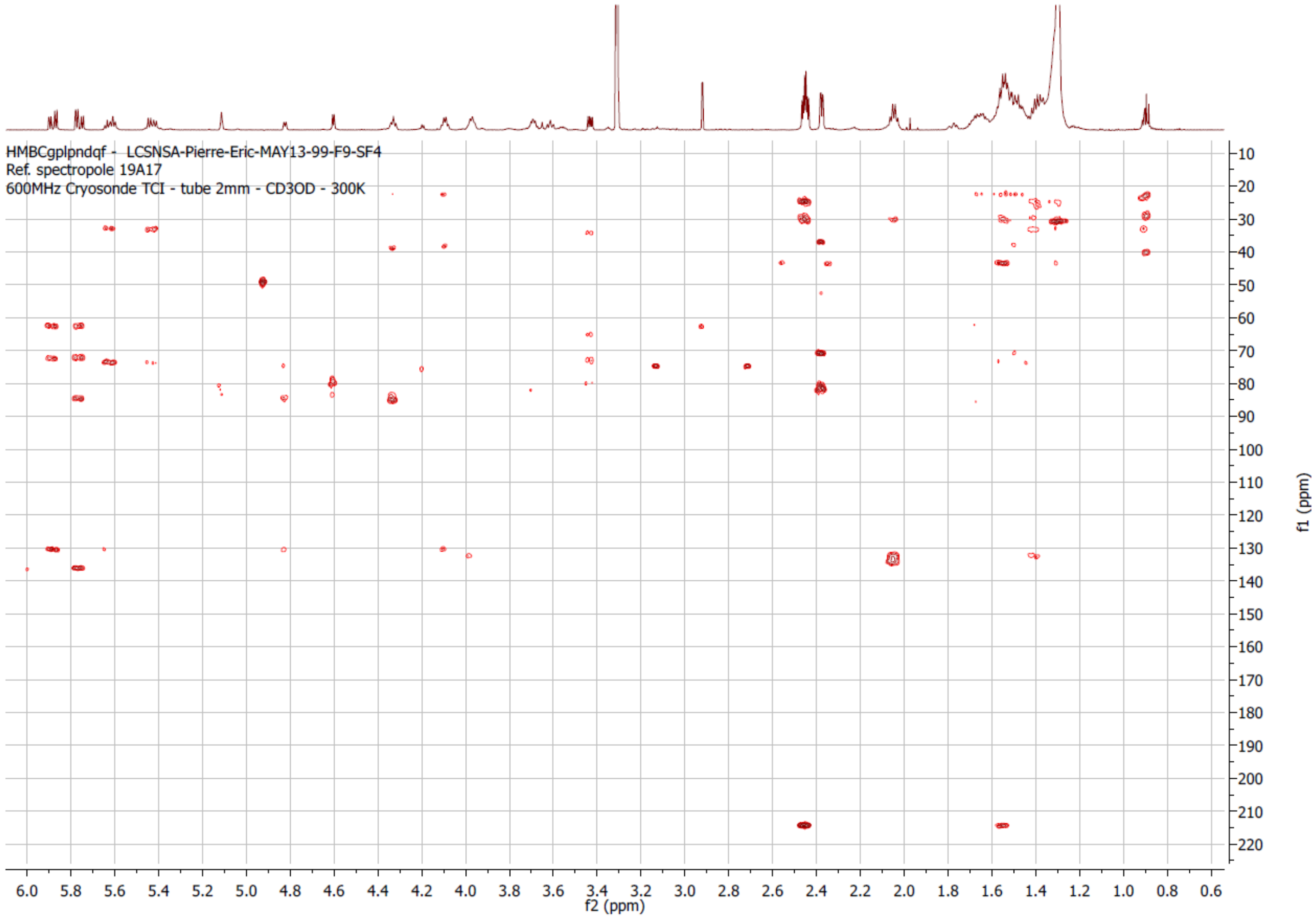

Figure S6: HRESIMS spectrum for osirisyne H (**2**)

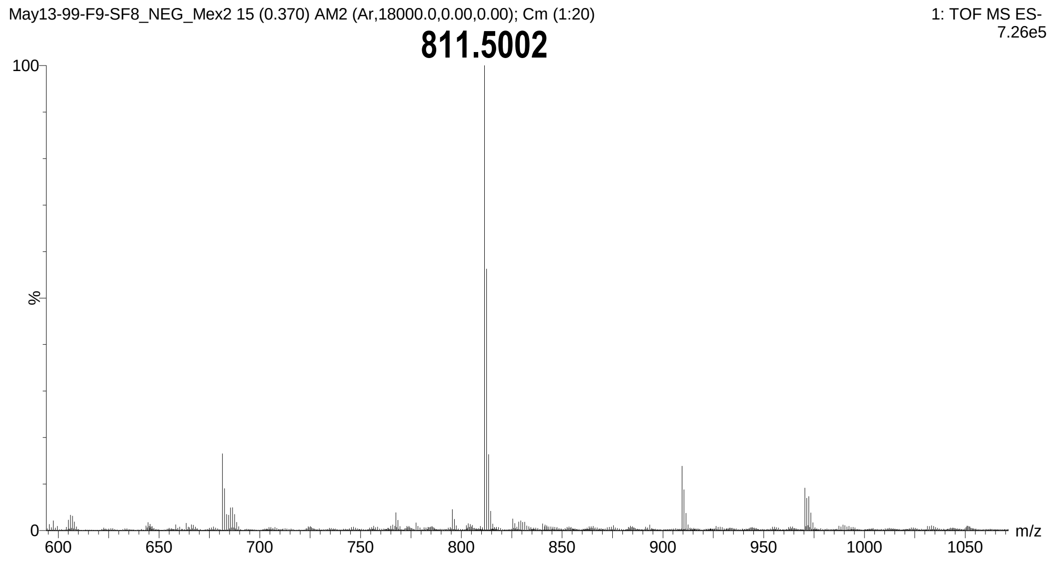

Figure S7: ESI⁺-MS/MS (S7.a.) and ESI⁻-MS/MS (S7.b.) spectra of osirisyne H (**2**) with outlines of dissociation of the precursor ion.

S7.a

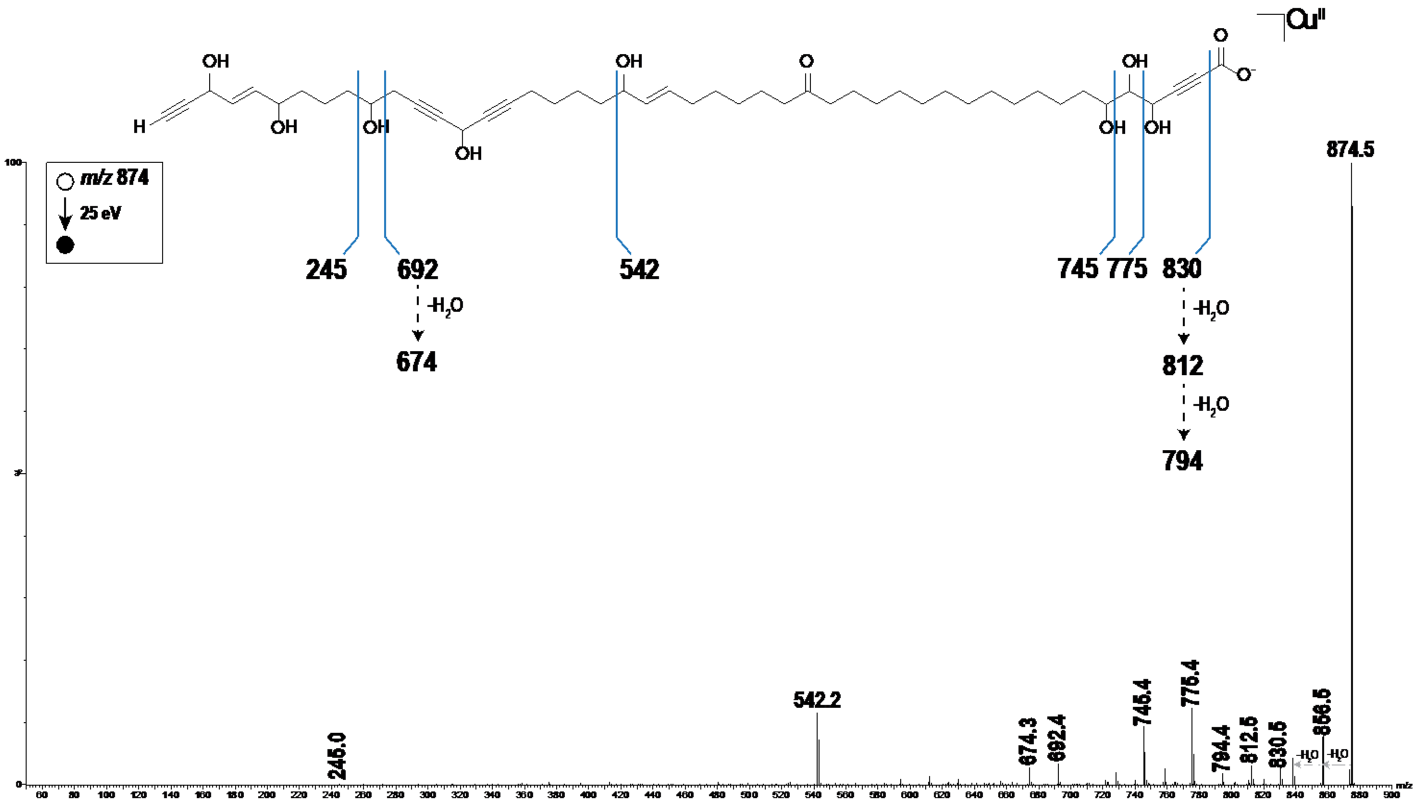

S7.b.

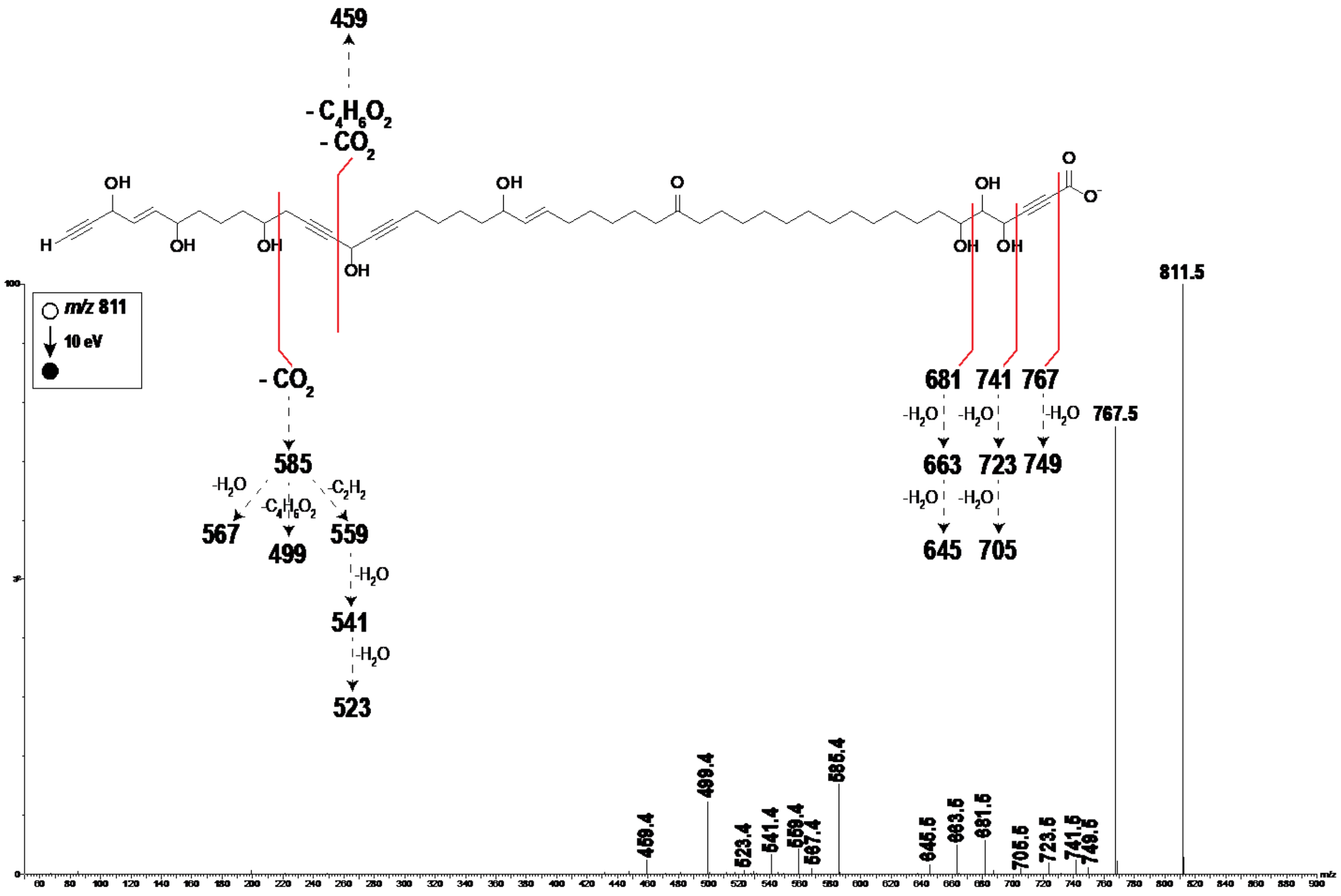

459
- $C_4H_6O_2$
- CO_2
OH
OH
OH
O
OH
O
O⁻
H
OH
OH
OH
OH
OH
811.5
m/z 811
10 eV
- CO_2
585
-H_2O
-$C_4H_6O_2$
-C_2H_2
567
499
559
-H_2O
541
-H_2O
523
681
741
767
-H_2O
-H_2O
-H_2O
767.5
663
723
749
-H_2O
-H_2O
645
705
459.4
499.4
523.4
541.4
559.4
567.4
585.4
645.5
663.5
681.5
705.5
723.5
741.5
749.5
m/z

Figure S8*:* Dissociation mechanisms of the fragmentation of osirisyne H (**2**) in ESI$^+$-MS/MS with the mass *m/z* of the different fragments.

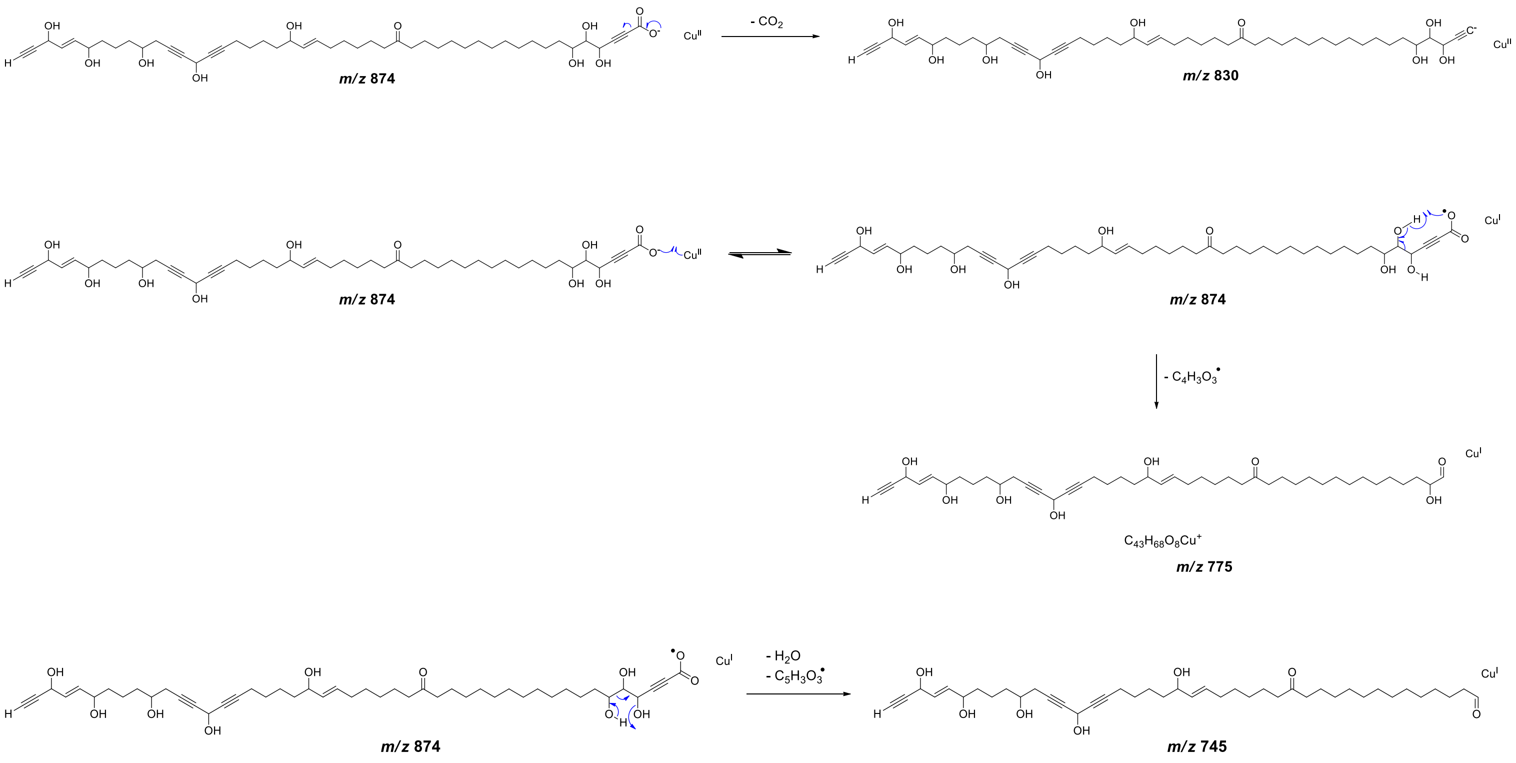

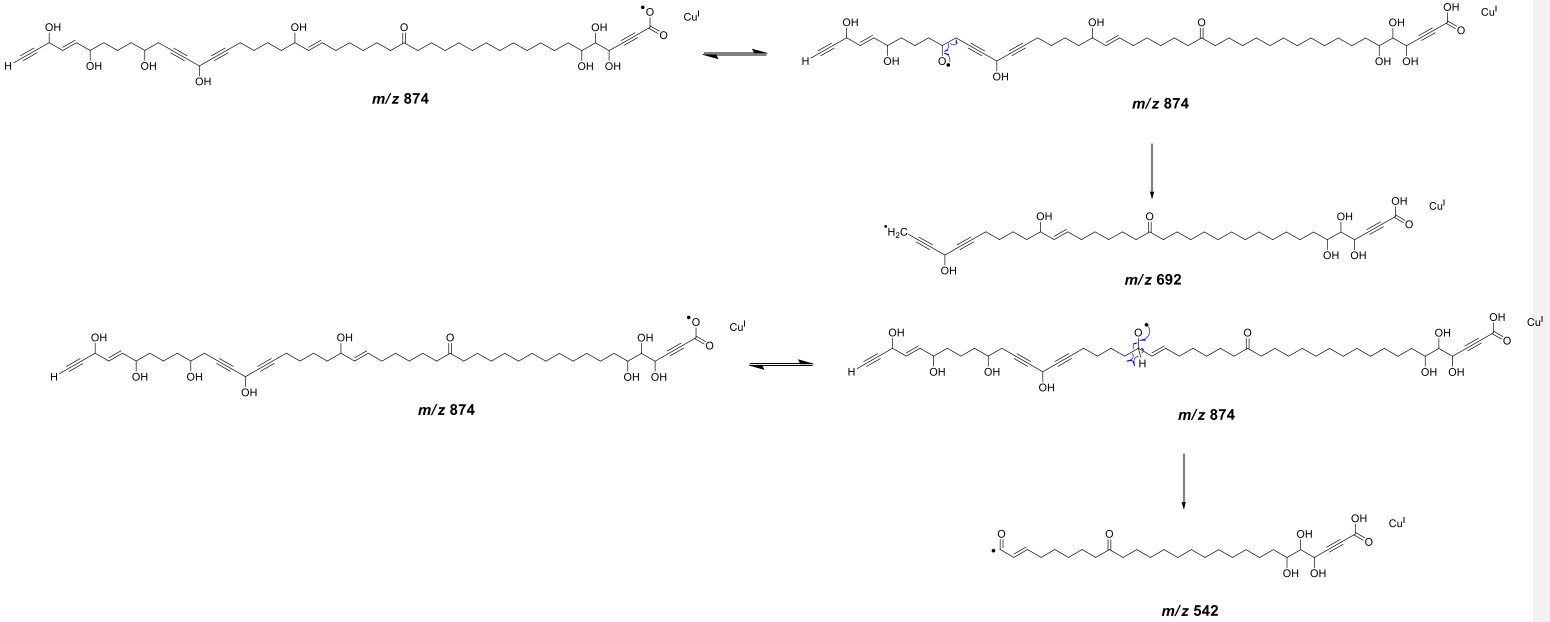
m/z 874
m/z 874
m/z 692
m/z 874
m/z 874
m/z 542
Cu^I
OH
O
H

Figure S9: ^{1}H NMR (600 MHz, CD_3OD) spectrum for osirisyne H (**2**)

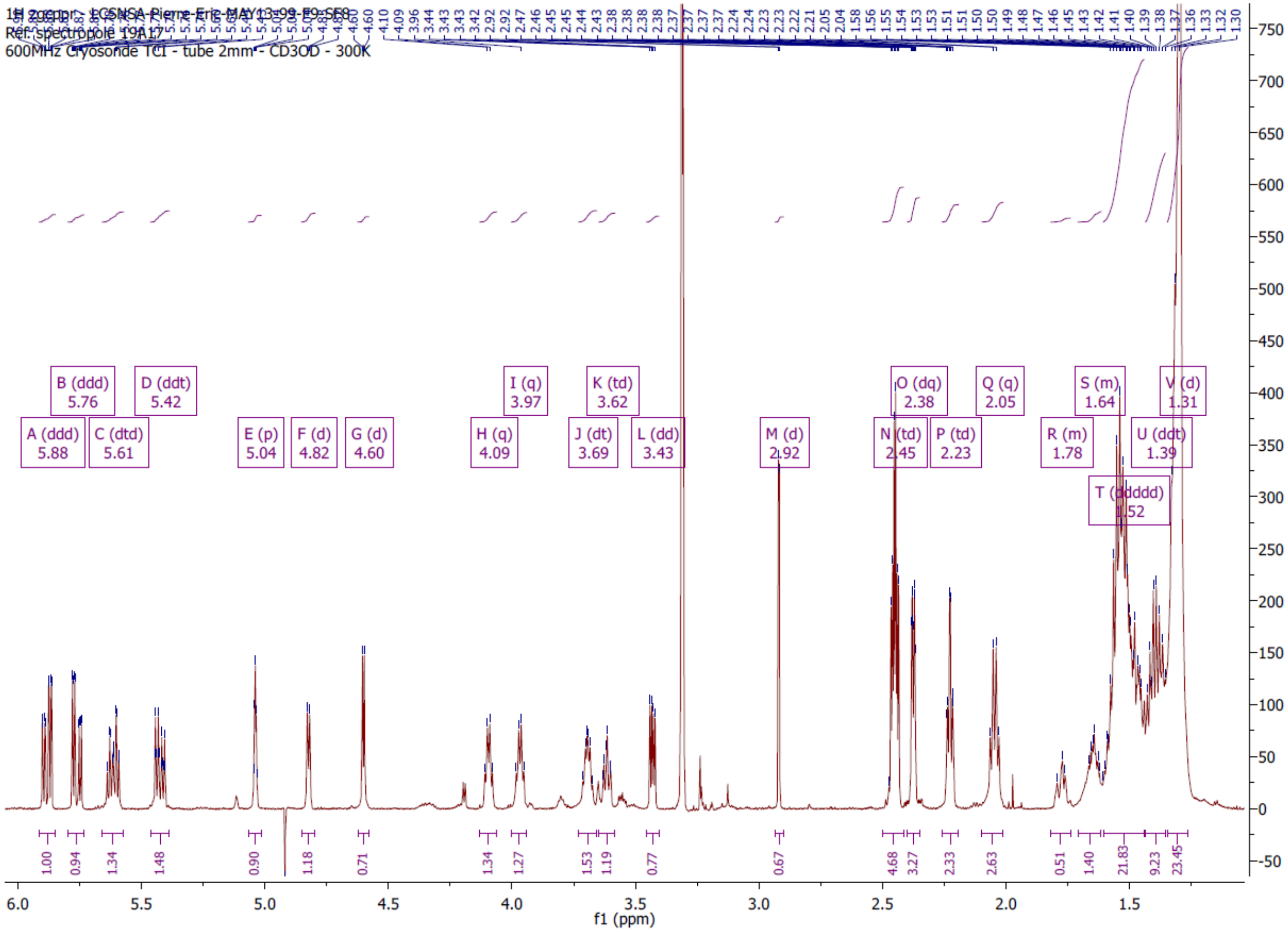

Figure S10: ^{1}H-^{1}H COSY NMR (600 MHz) spectrum for osirisyne H (**2**)

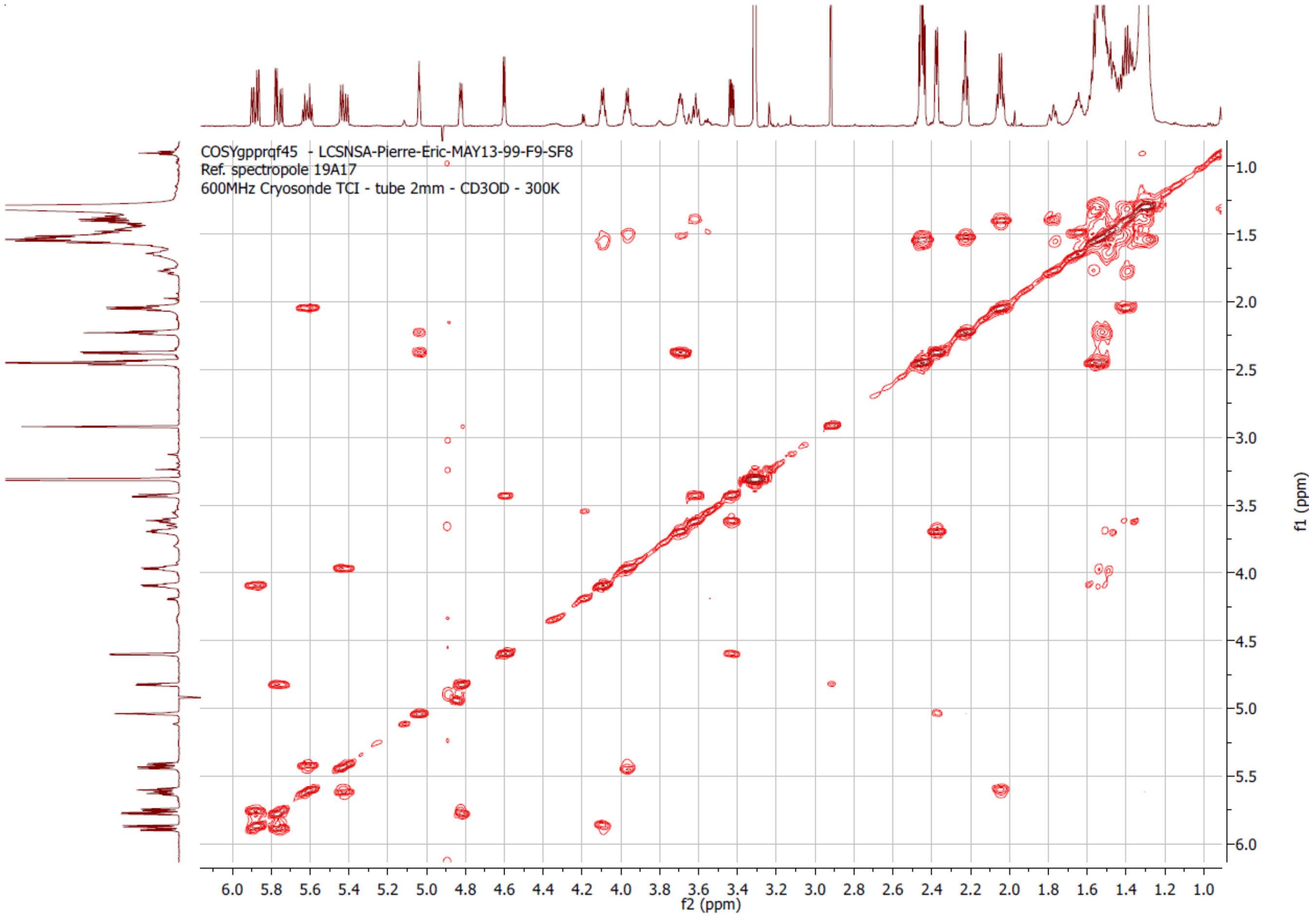

Figure S11: ^{1}H-^{13}C HSQC NMR (600 MHz) spectrum for osirisyne H (**2**)

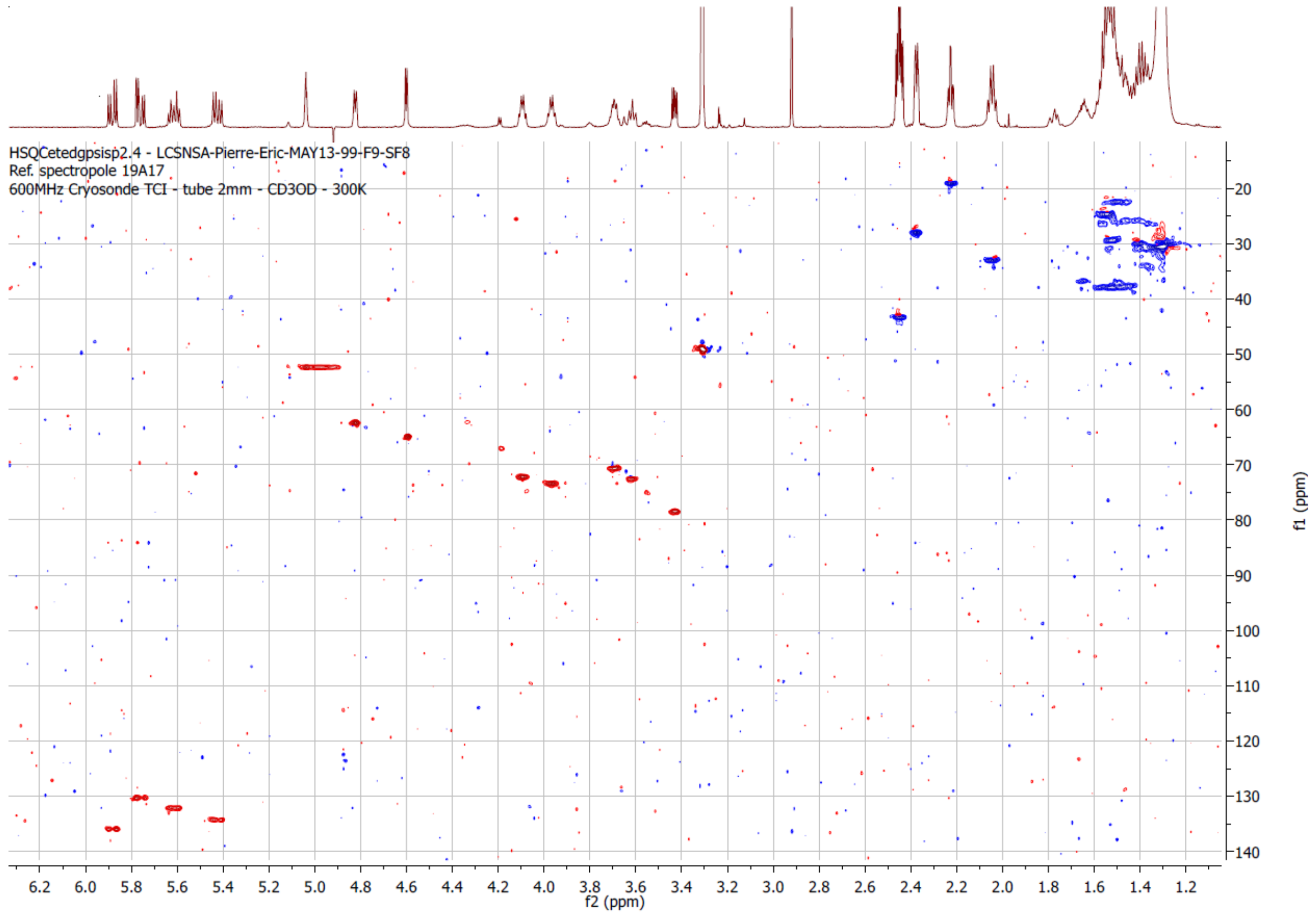

Figure S12: ^{1}H-^{13}C HMBC NMR (600 MHz) spectrum for osirisyne H (**2**)

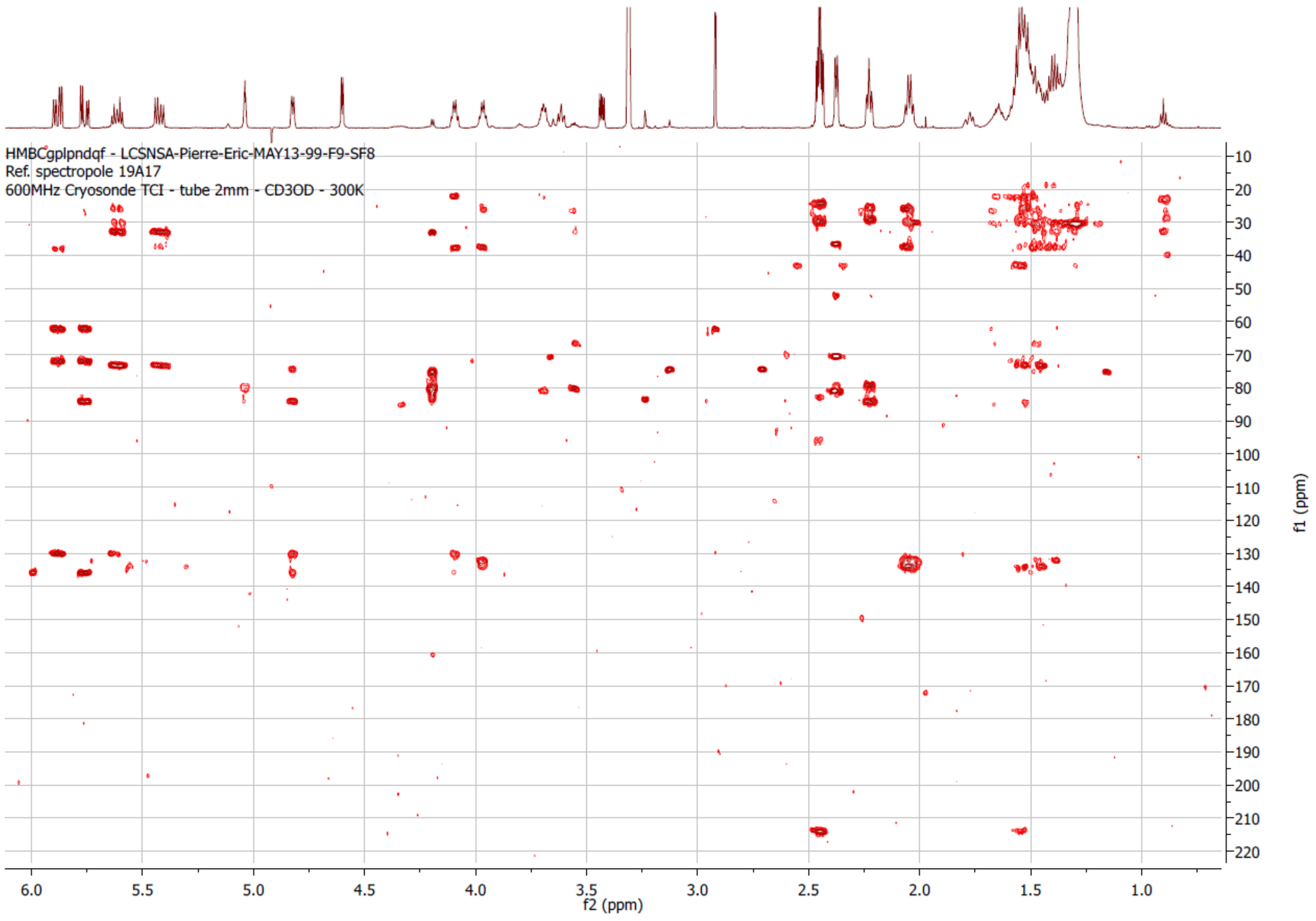

Figure S13: ^{1}H-^{13}C HSQC-TOCSY NMR (600 MHz) spectrum for osirisyne H (**2**)

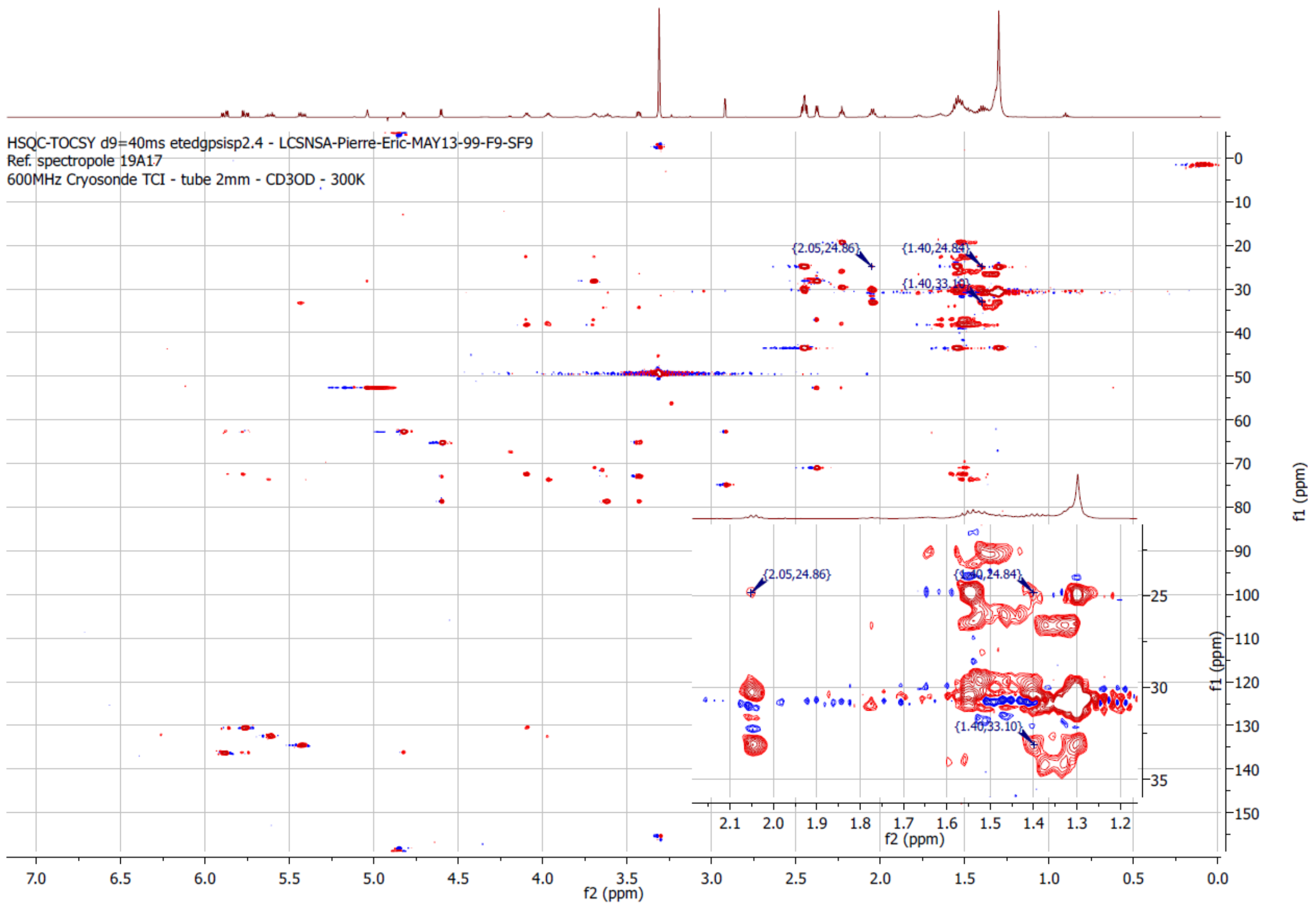

Figure S14: HRESIMS spectrum for osirisyne I (**3**)

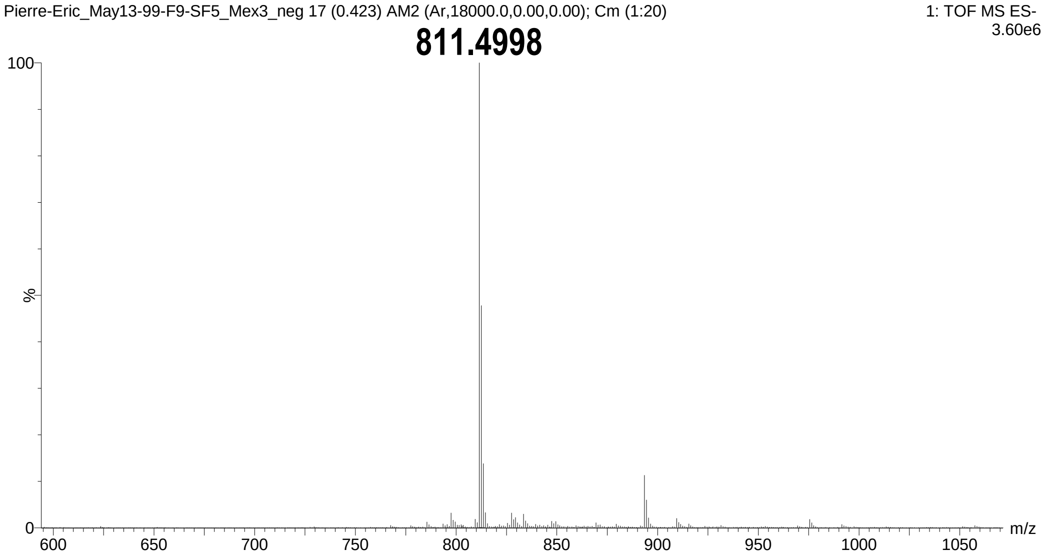

Figure S15*:* ESI+-MS/MS (S13.a.) and ESI--MS/MS (S13.b.) spectra of osirisyne I (**3**) with outlines of dissociation of the precursor ion.

S13.a.

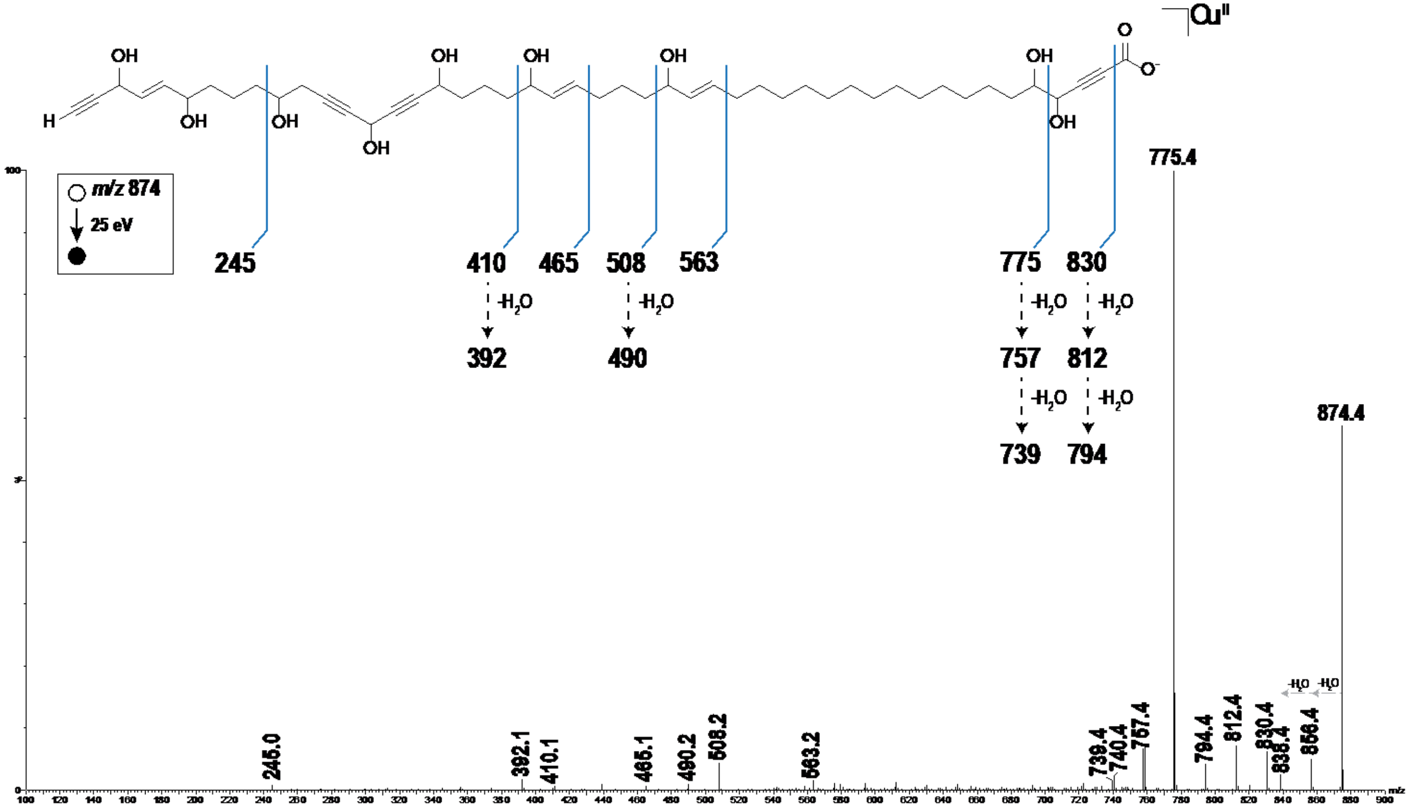

S13.b.

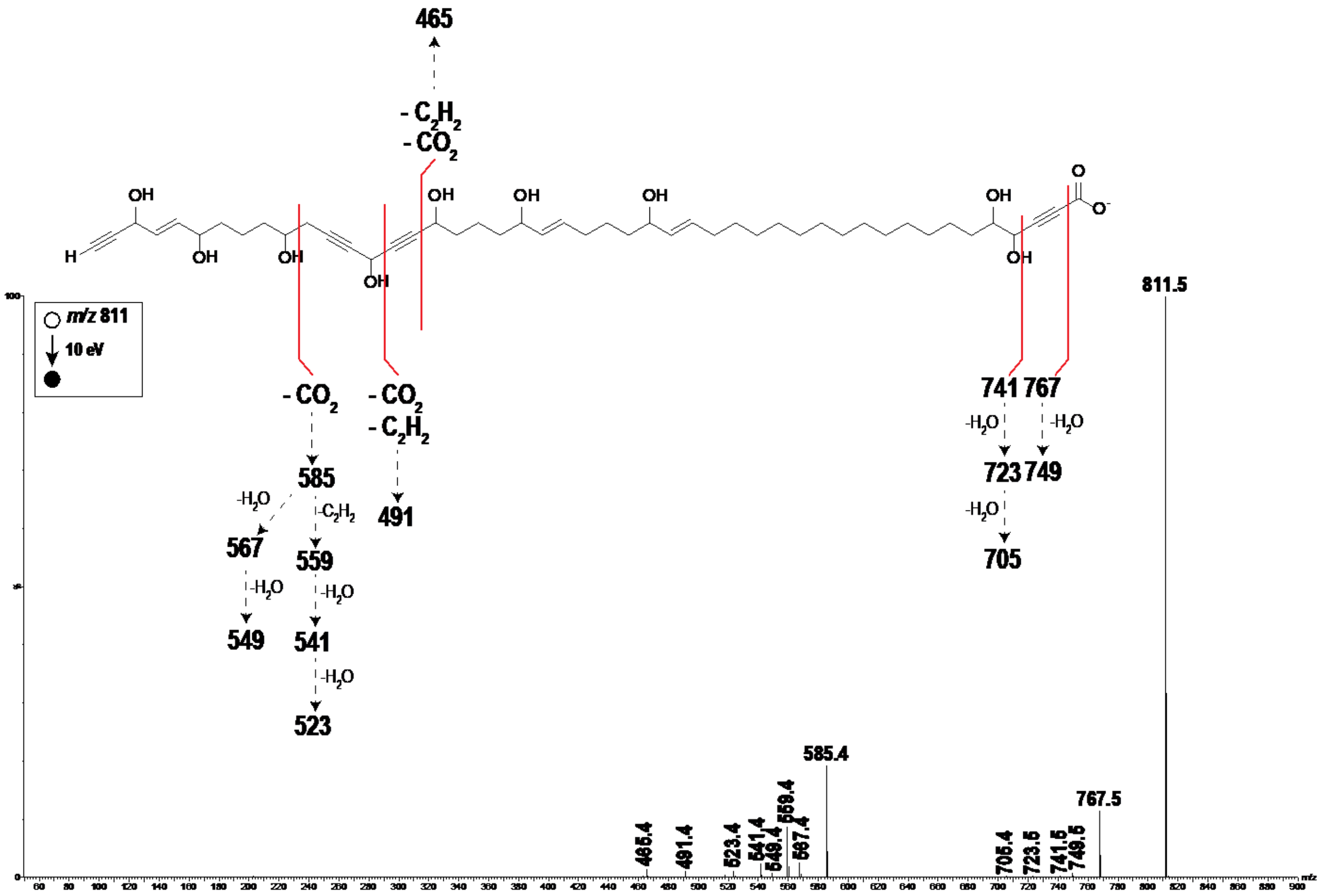

465
- C_2H_2
- CO_2
OH
OH
OH
OH
OH
O
O⁻
H
OH
OH
OH
OH
811.5
m/z 811
10 eV
- CO_2
- CO_2
- C_2H_2
741 767
-H_2O
-H_2O
723 749
-H_2O
705
585
-H_2O
-C_2H_2
491
567
559
-H_2O
-H_2O
549
541
-H_2O
523
585.4
767.5
465.4
491.4
523.4
541.4
549.4
559.4
567.4
705.4
723.5
741.5
749.5
m/z

Figure S16*:* Dissociation mechanisms of the fragmentation of osirisyne I (**3**) in ESI$^+$-MS/MS (14.a.) and ESI$^-$-MS/MS (14.b.) with the mass *m/z* of the different fragments.

14.a.

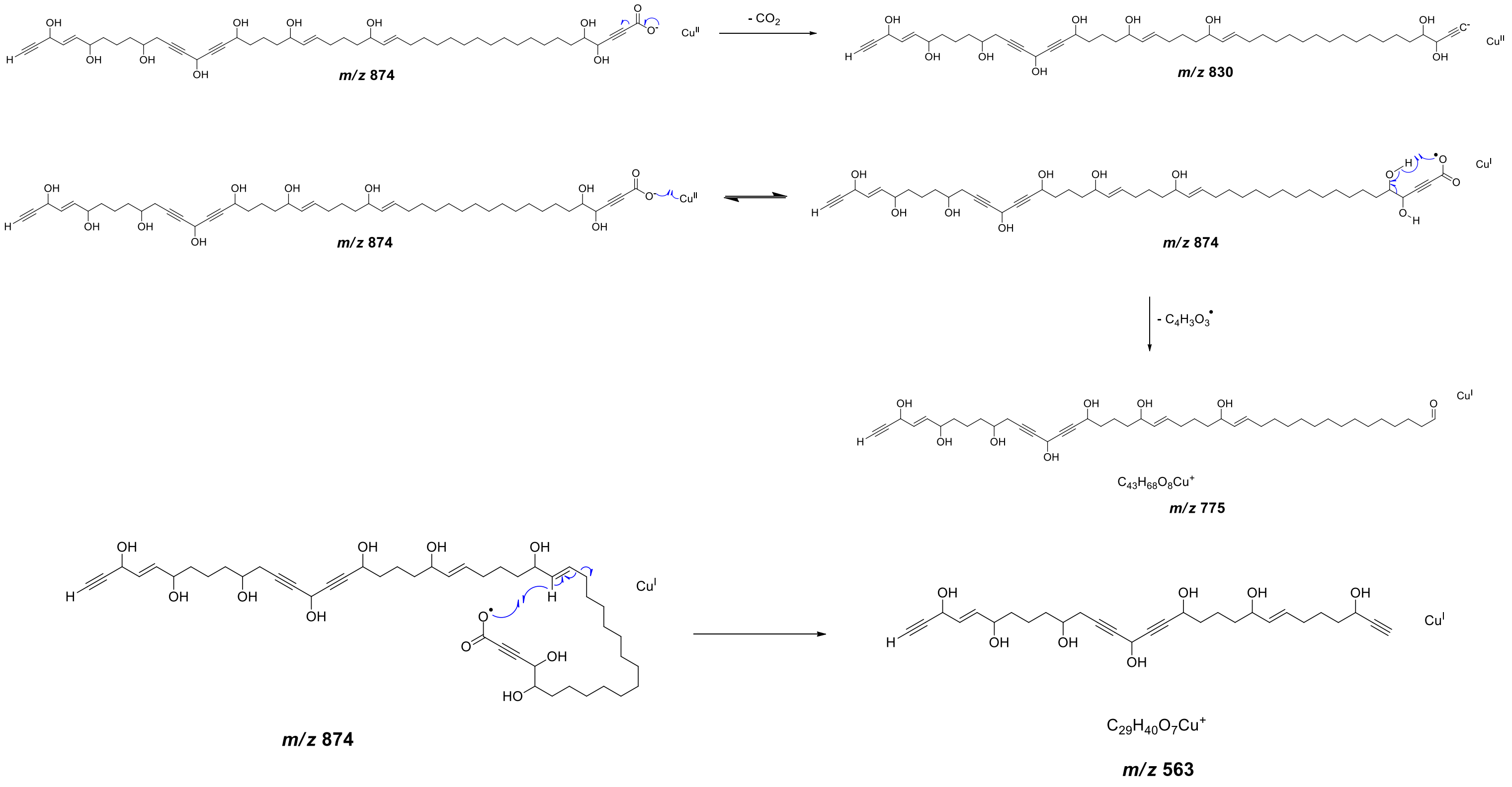

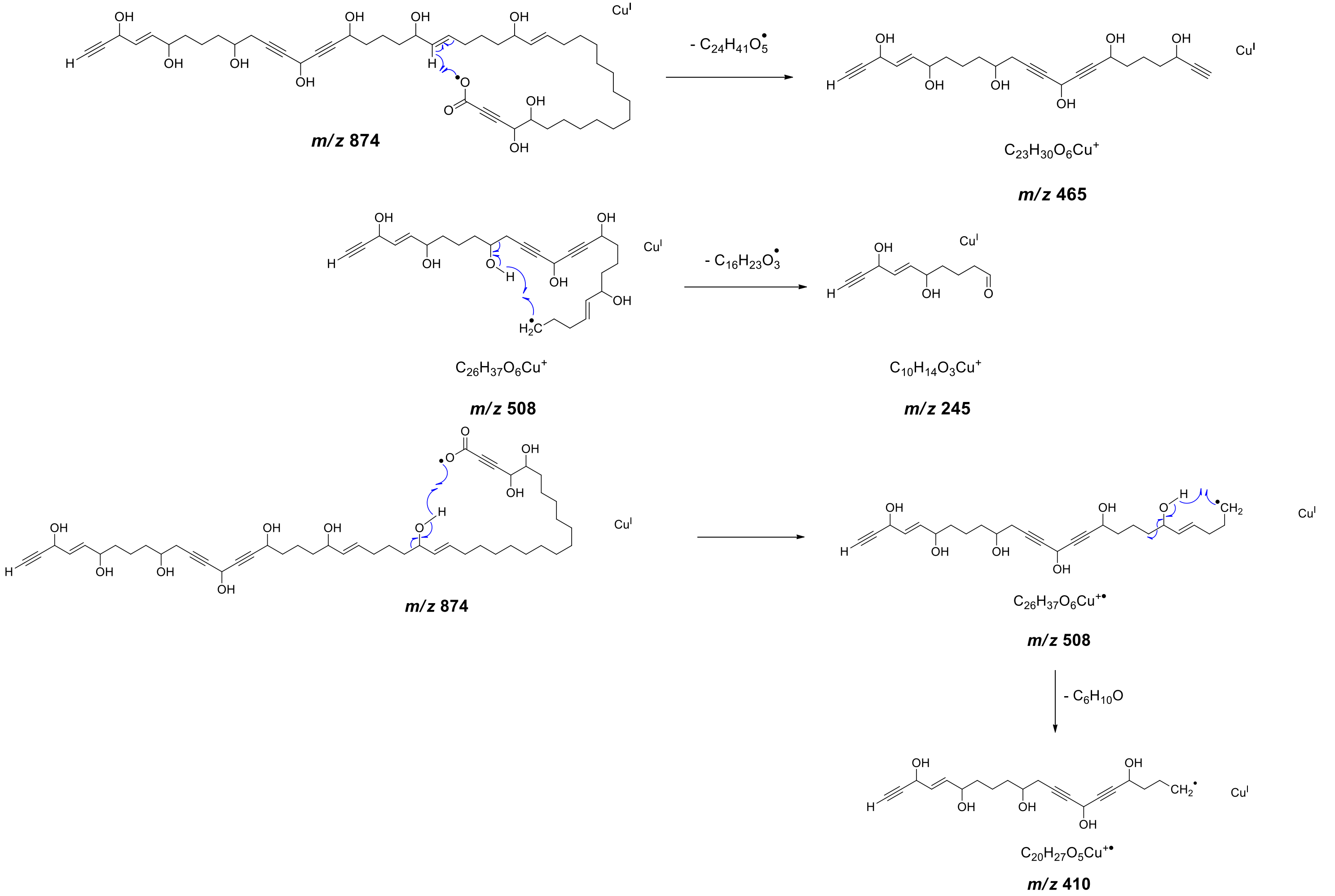

m/z 874
- $C_{24}H_{41}O_5^{\bullet}$
$C_{23}H_{30}O_6Cu^{+}$
m/z 465
$C_{26}H_{37}O_6Cu^{+}$
m/z 508
- $C_{16}H_{23}O_3^{\bullet}$
$C_{10}H_{14}O_3Cu^{+}$
m/z 245
m/z 874
$C_{26}H_{37}O_6Cu^{+\bullet}$
m/z 508
- $C_6H_{10}O$
$C_{20}H_{27}O_5Cu^{+\bullet}$
m/z 410

14.b.

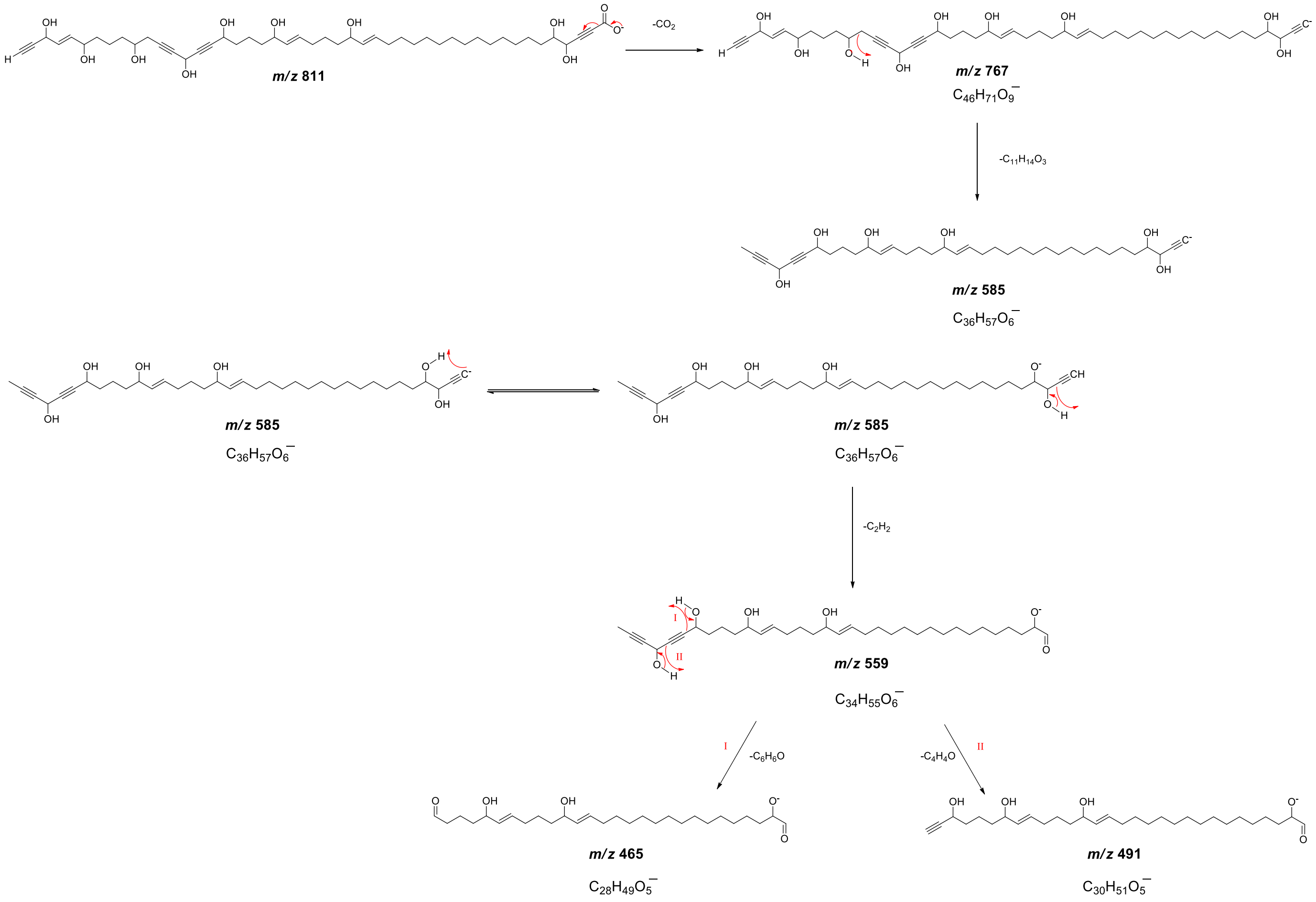
m/z 811
-CO2
m/z 767
C46H71O9−
-C11H14O3
m/z 585
C36H57O6−
m/z 585
C36H57O6−
m/z 585
C36H57O6−
-C2H2
m/z 559
C34H55O6−
I
-C6H6O
II
-C4H4O
m/z 465
C28H49O5−
m/z 491
C30H51O5−

Figure S17: IRTF spectrum for osirisyne I (**3**)

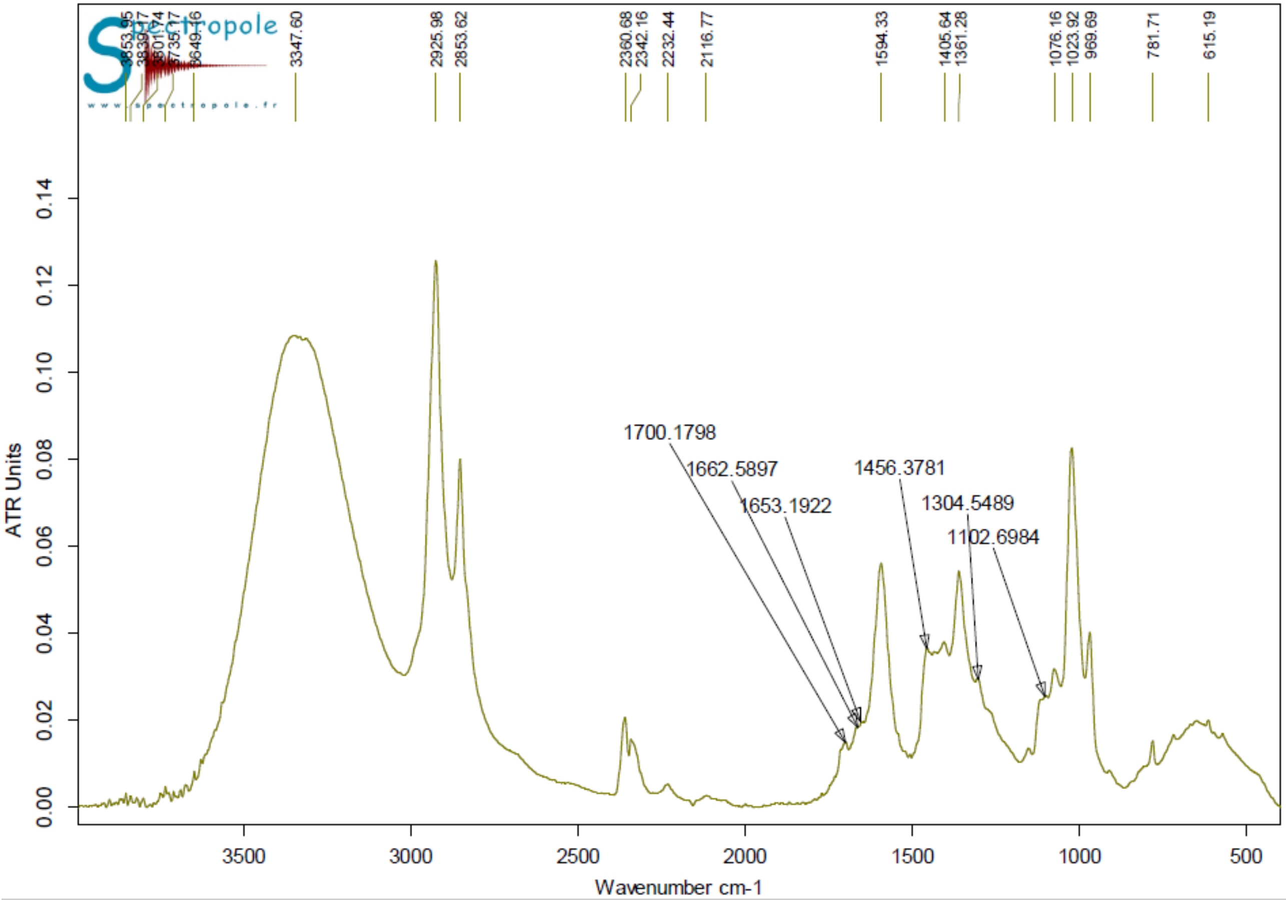

Figure S18: ^{1}H NMR (600 MHz, CD_3OD) spectrum for osirisyne I (**3**)

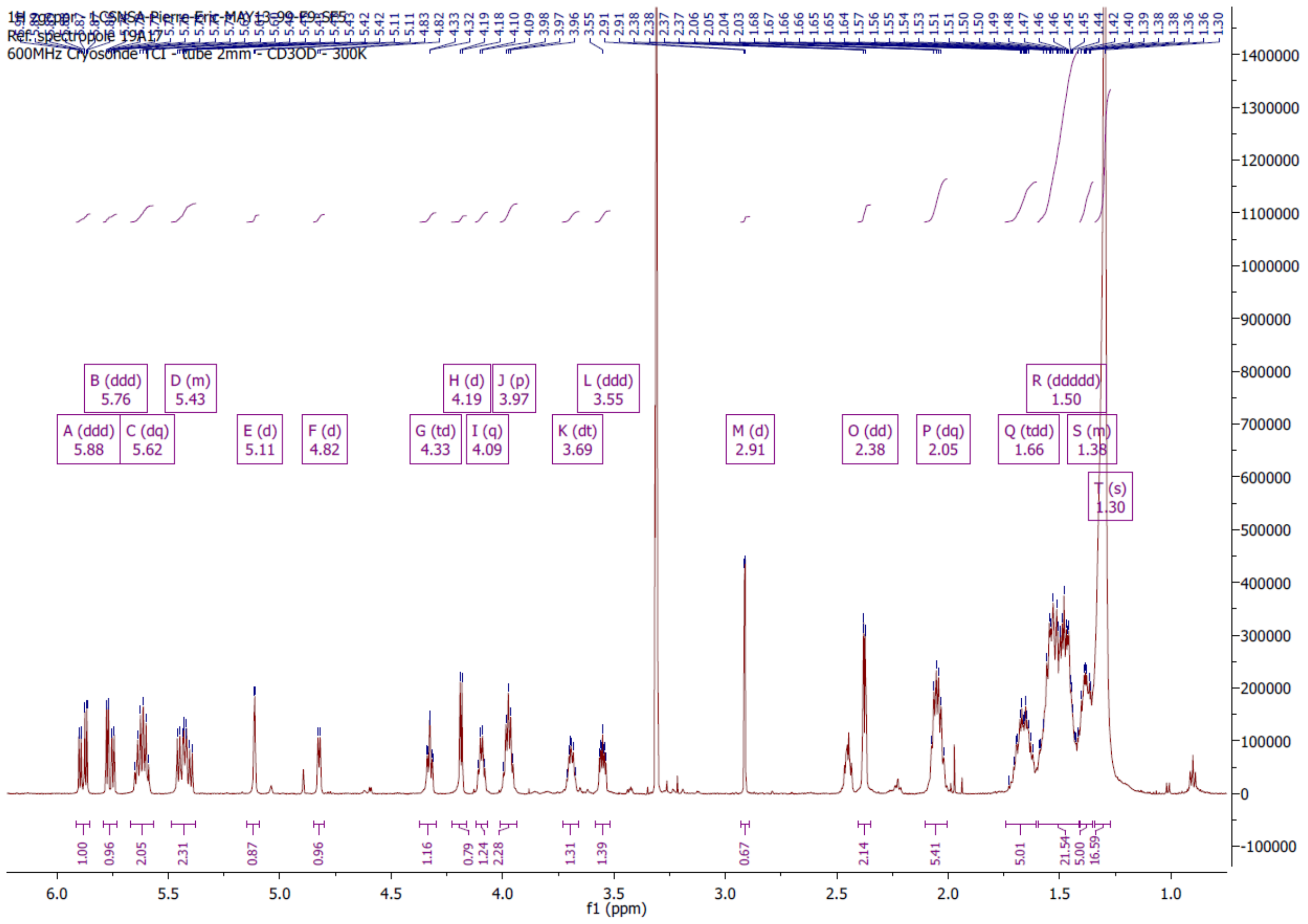

Figure S19: ^{13}C NMR (600 MHz, CD_3OD) spectrum for osirisyne I (**3**)

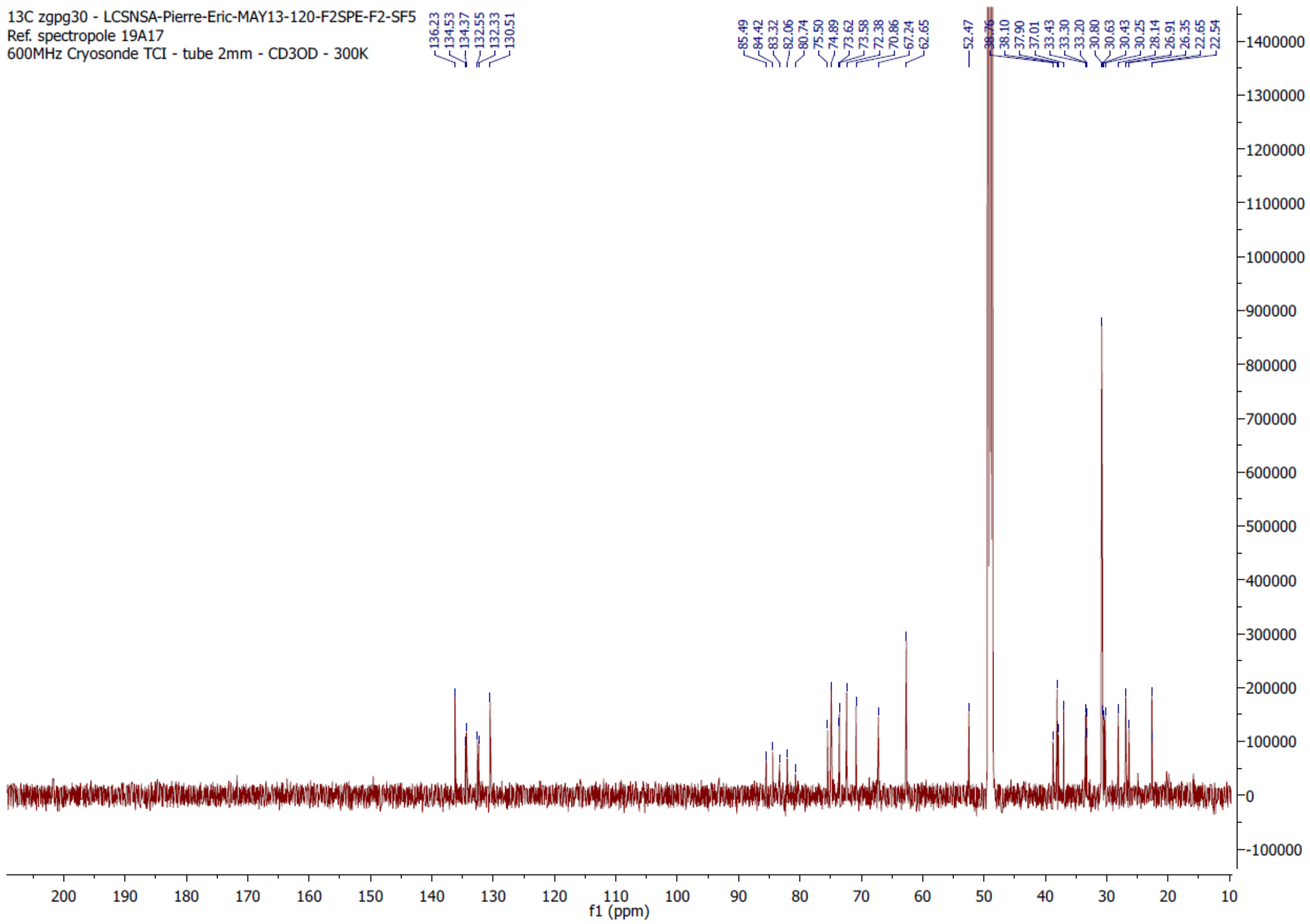

Figure S20: ^{1}H-^{1}H COSY NMR (600 MHz) spectrum for osirisyne I (**3**)

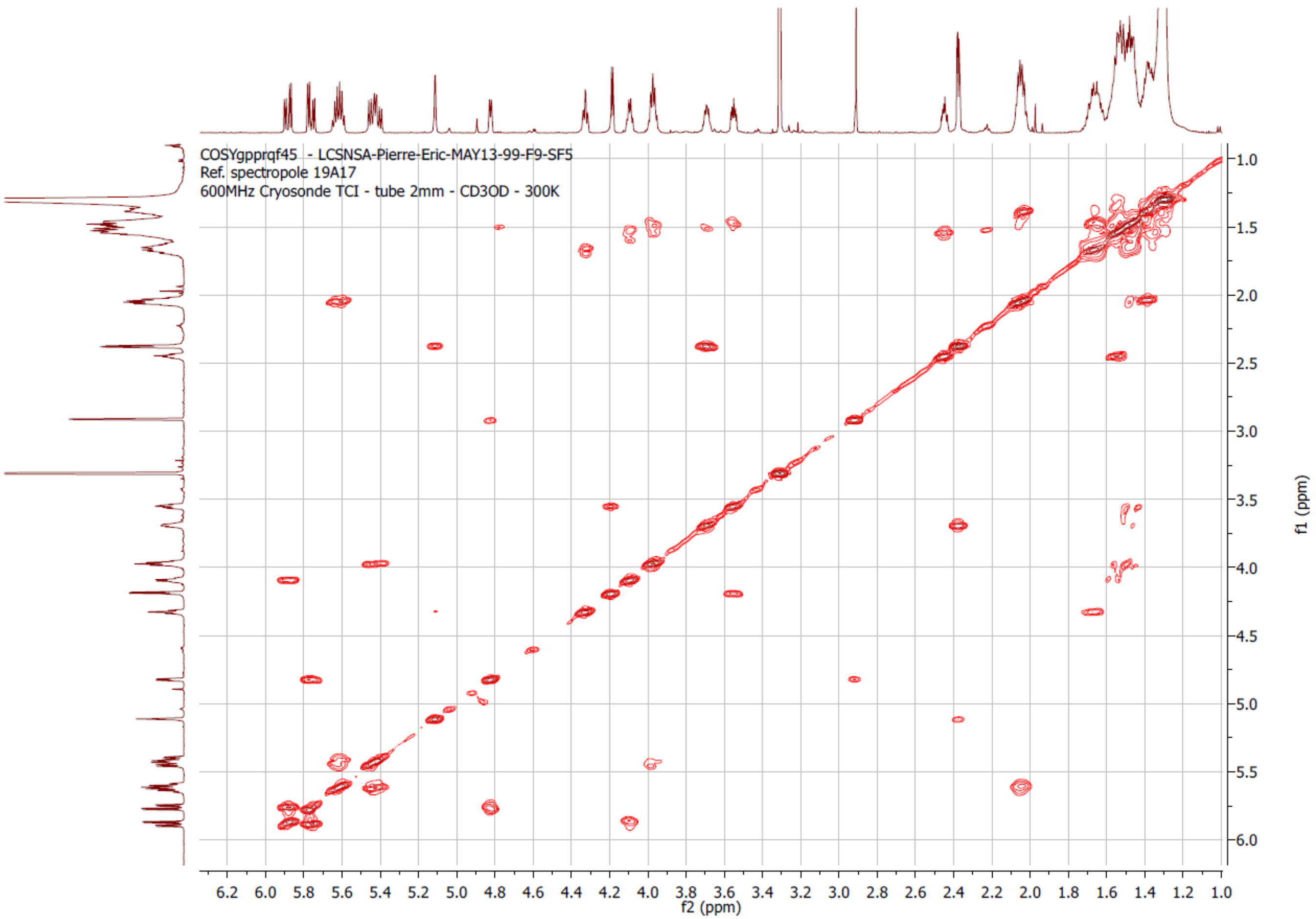

Figure S21: ^{1}H-^{13}C HSQC NMR (600 MHz) spectrum for osirisyne I (**3**)

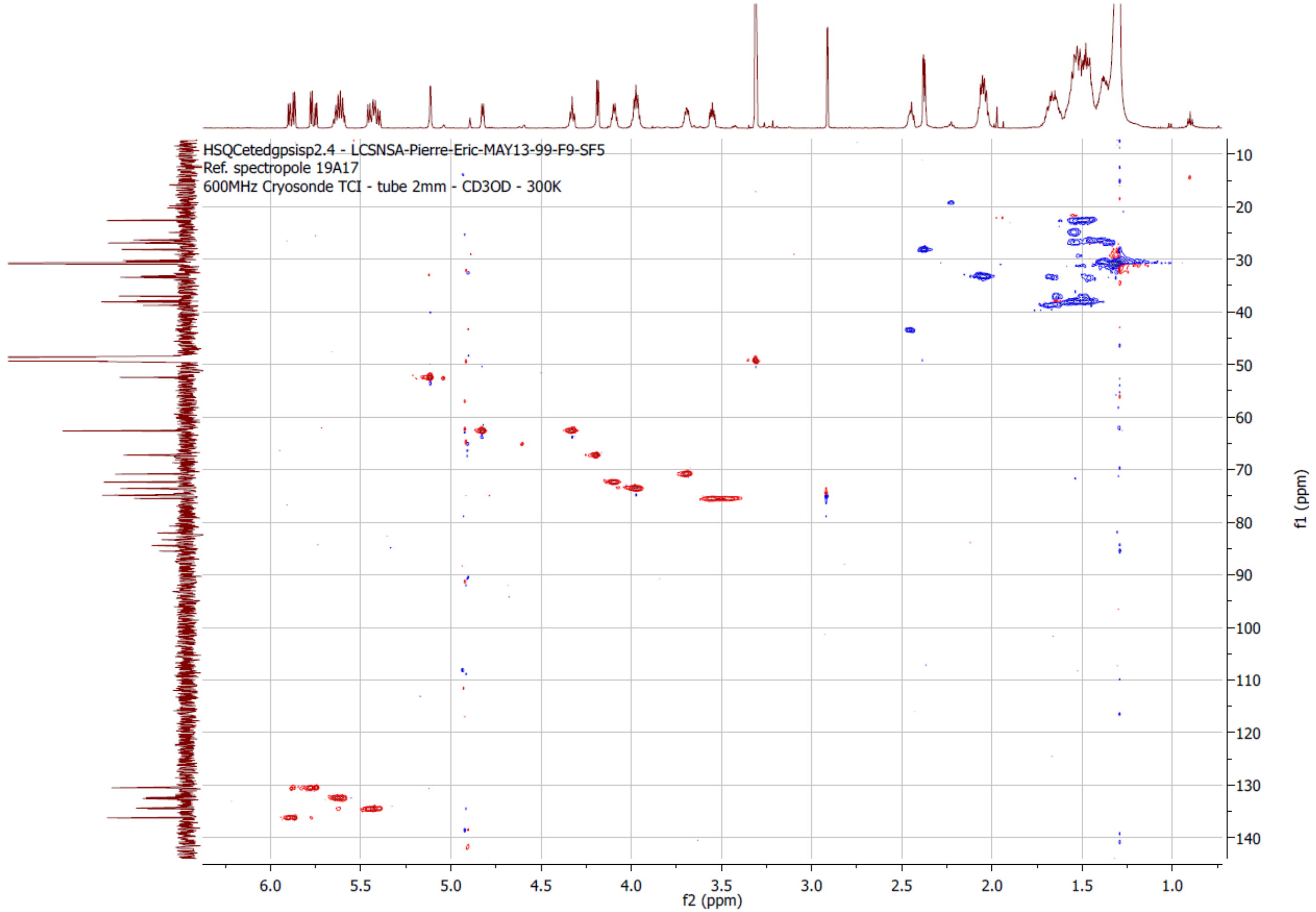

Figure S22: ^{1}H-^{13}C HMBC NMR (600 MHz) spectrum for osirisyne I (**3**)

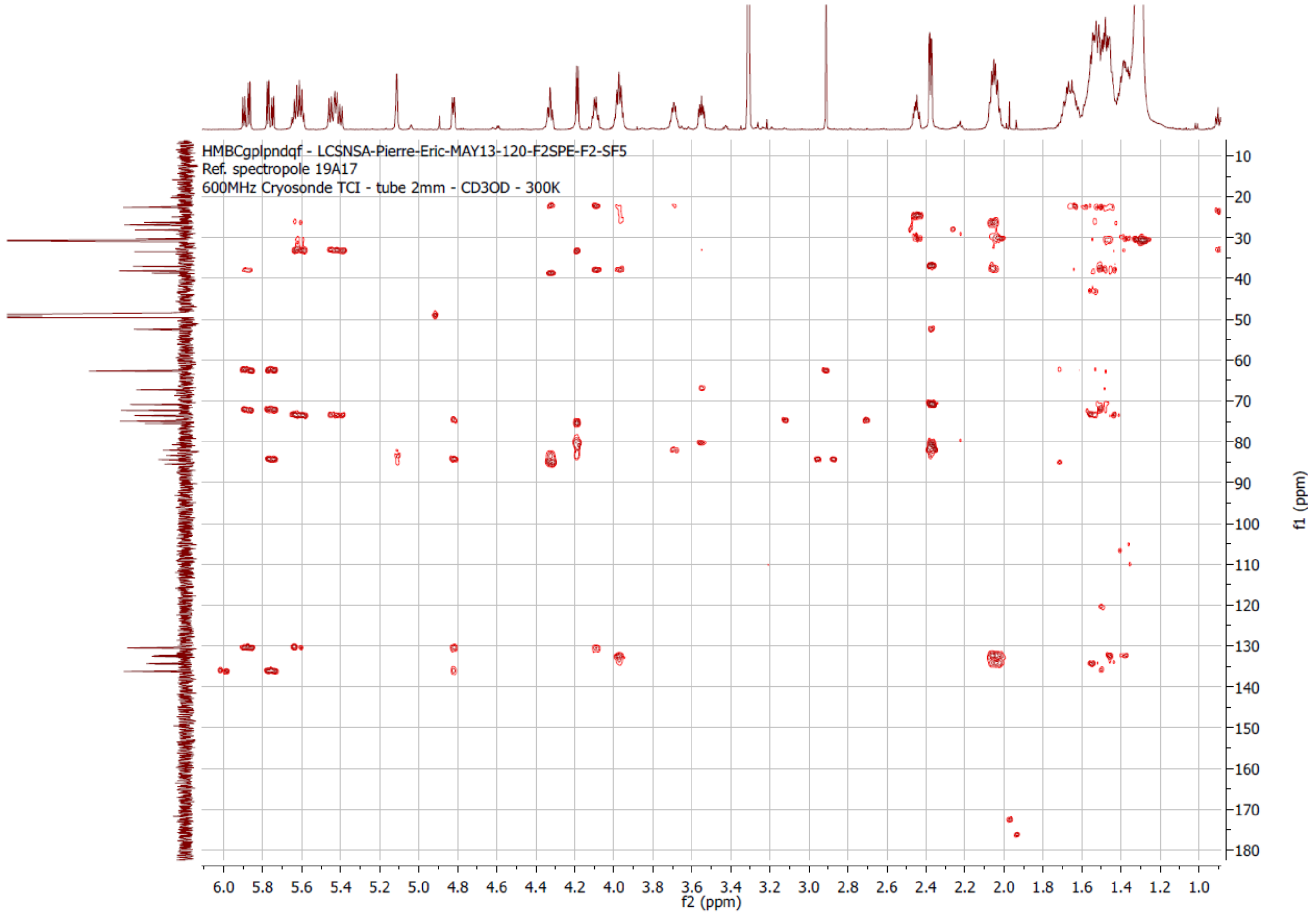

Figure S23: ^{1}H-^{1}H NOESY NMR (600 MHz) spectrum for osirisyne I (**3**)

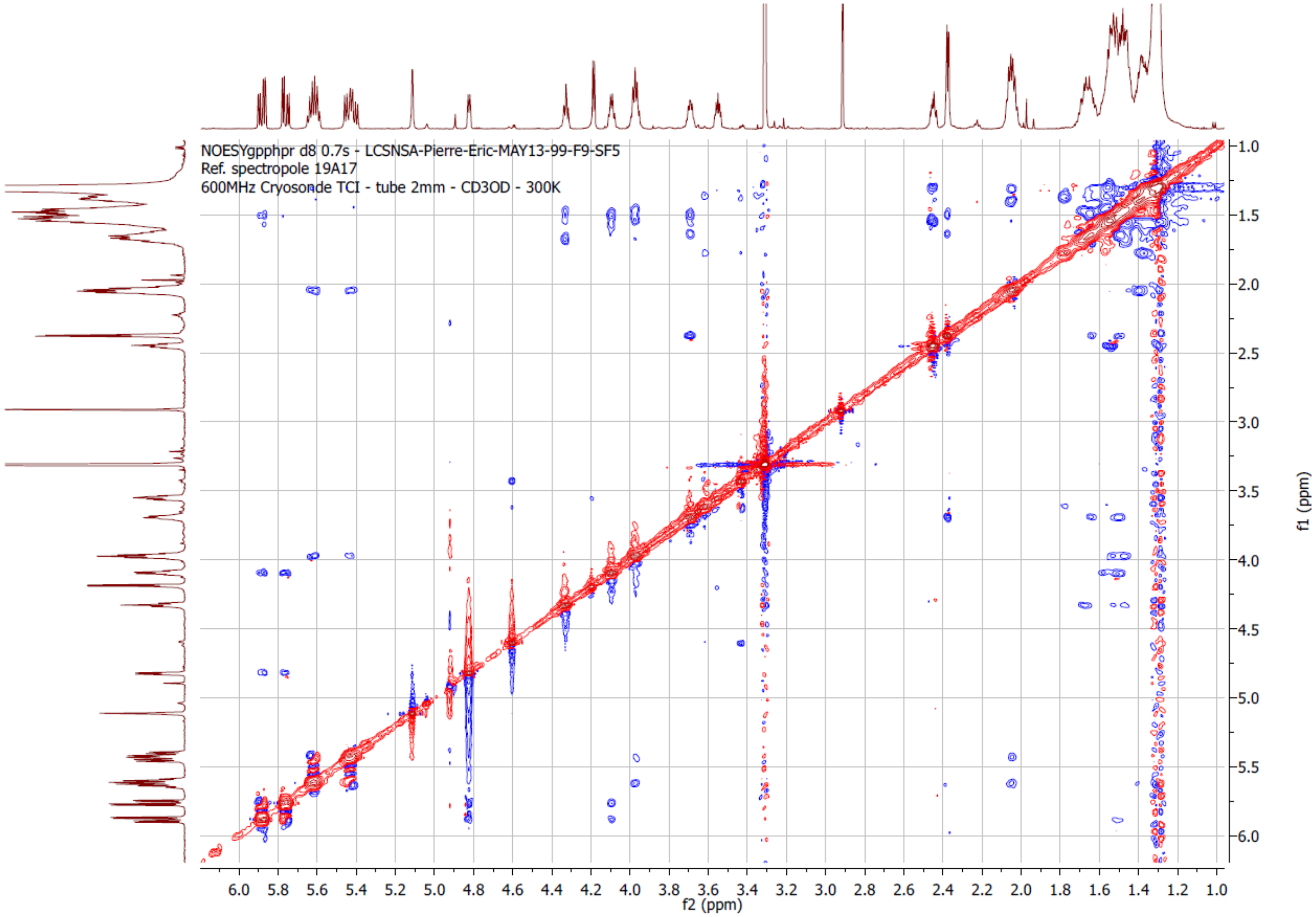

Figure S24: ^{1}H-^{13}C TOCSY-HSQC NMR (600 MHz) spectrum for osirisyne I (**3**)

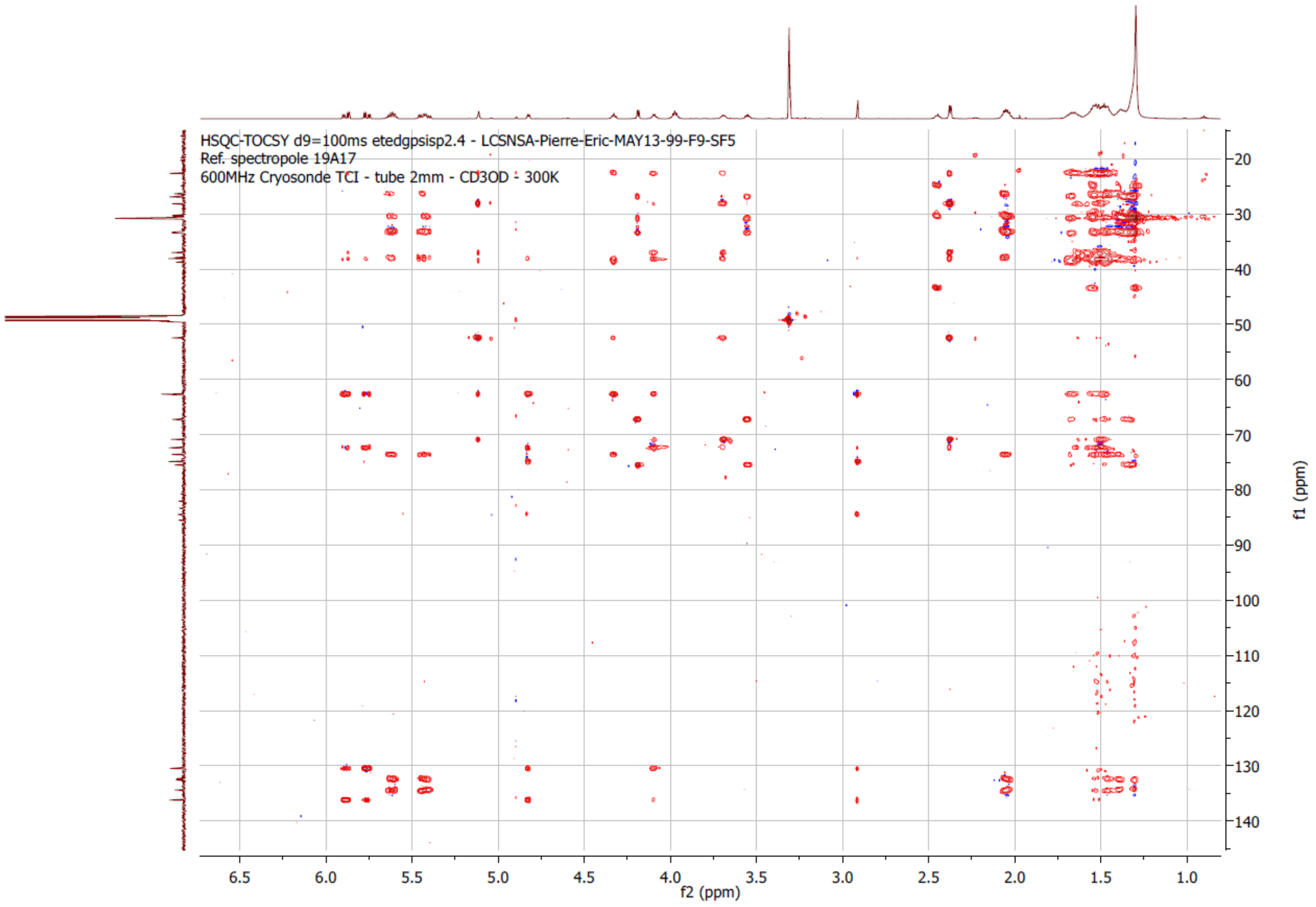

Figure S25: HRESIMS spectrum for osirisyne A (**4**)

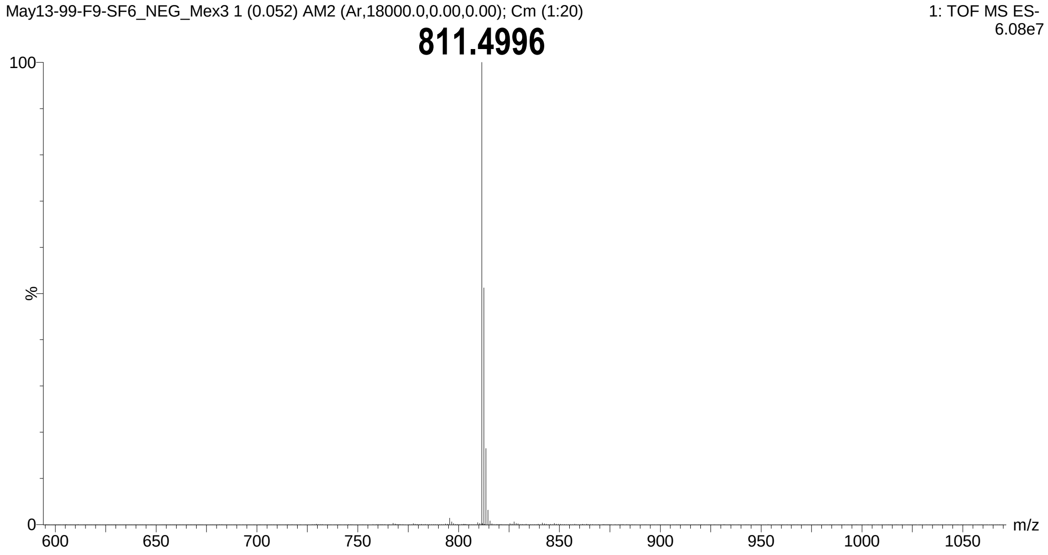

Figure S26*:* ESI+-MS/MS (S22.a.) and ESI–-MS/MS (S22.b.) spectra of osirisyne A (**4**) with outlines of dissociation of the precursor ion.

S22.a.

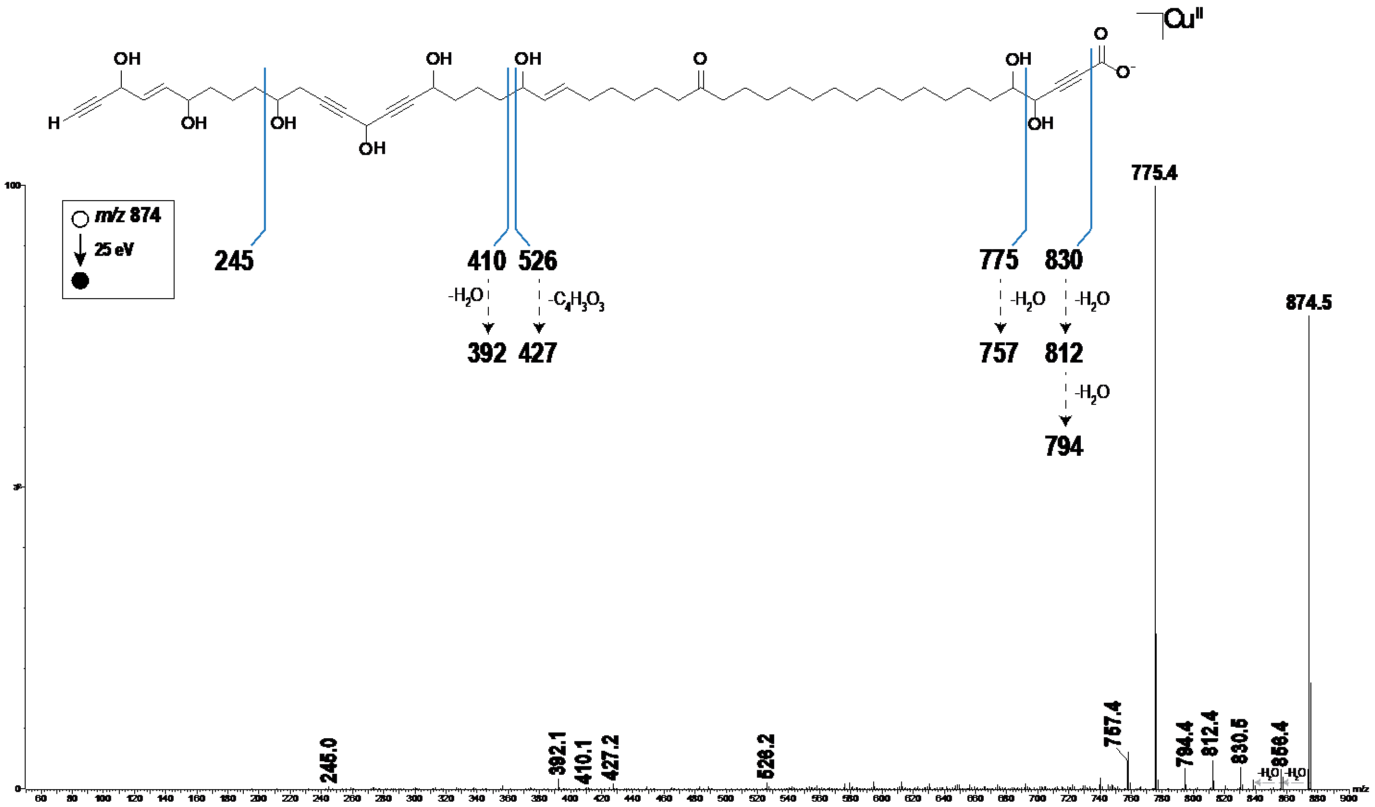

S22.b.

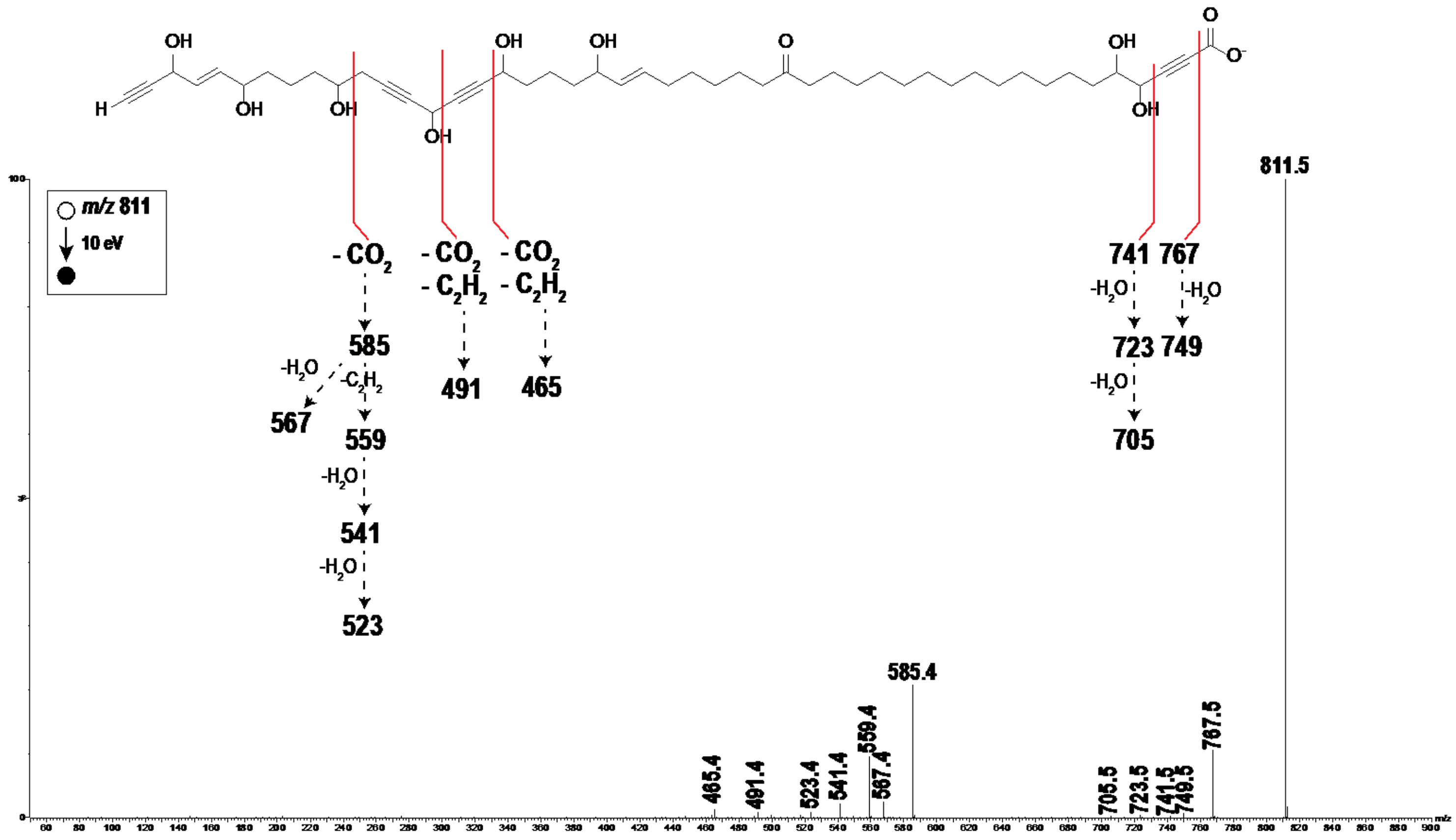

OH
OH
OH
OH
OH
O
OH
OH
O
O-
H
OH
m/z 811
10 eV
- CO2
- CO2
- C2H2
- CO2
- C2H2
585
-H2O
-C2H2
567
559
-H2O
541
-H2O
523
491
465
741 767
-H2O
-H2O
723 749
-H2O
705
811.5
585.4
465.4
491.4
523.4
541.4
559.4
567.4
705.5
723.5
741.5
749.5
767.5
m/z

Figure S27: ^{1}H NMR (600 MHz, CD_3OD) spectrum for osirisyne A (**4**)

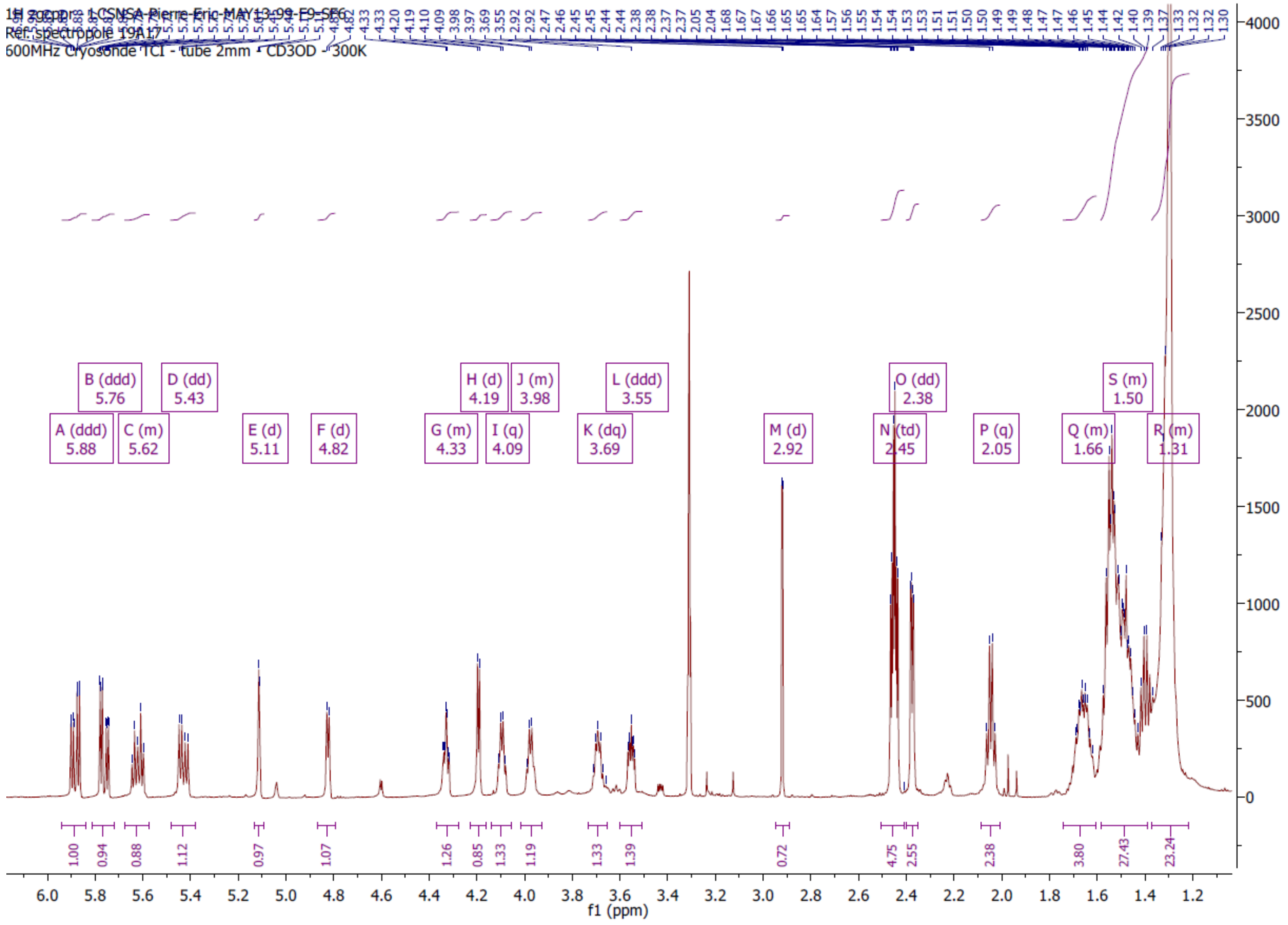

Figure S28: ^{13}C NMR (600 MHz, CD_3OD) spectrum for osirisyne A (**4**)

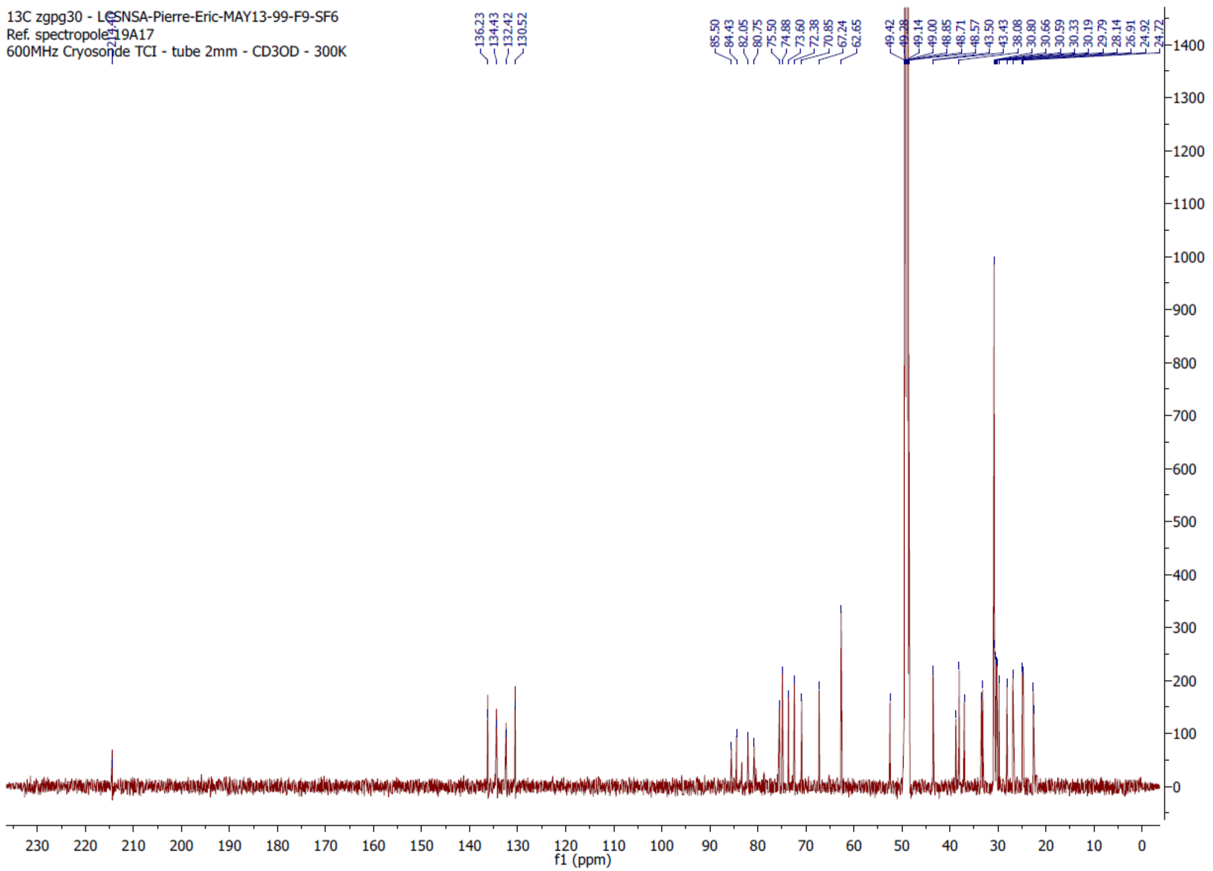

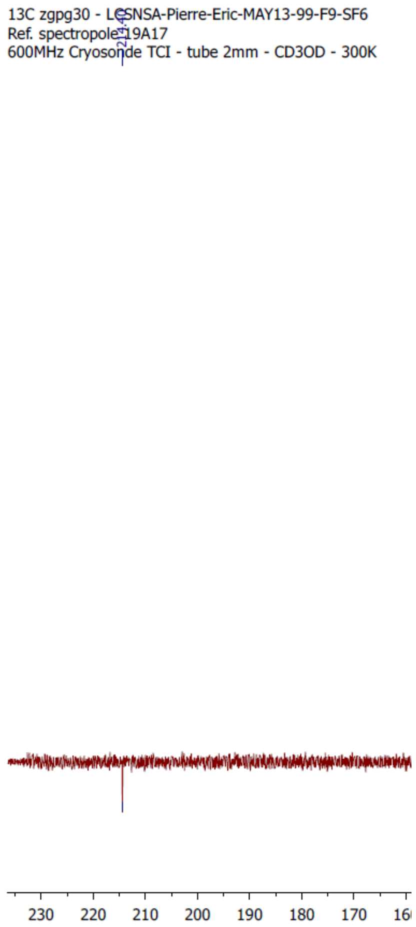

Deleted:

Figure S29: HRESIMS spectrum for osirisyne B (**4**)

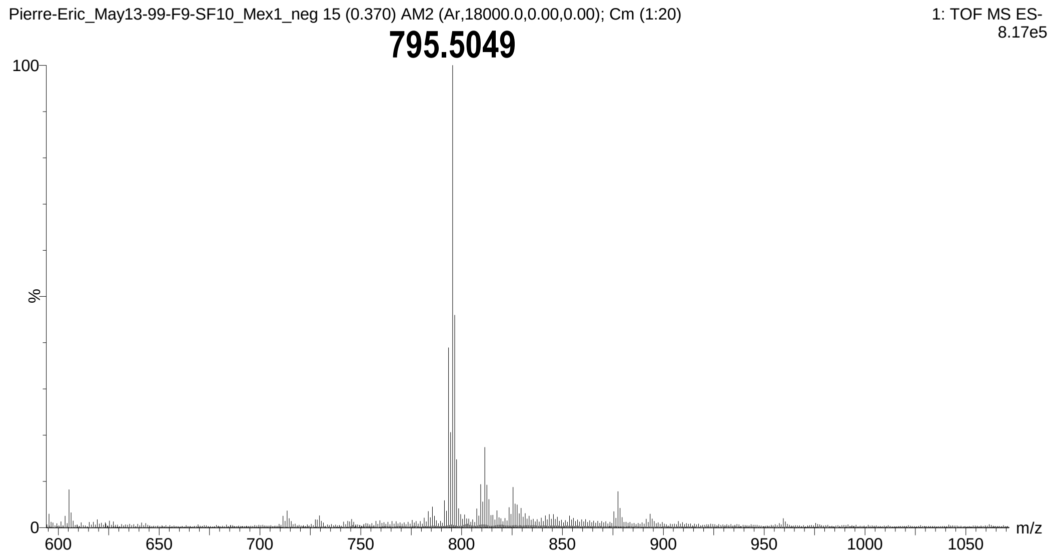

Figure S30: ESI$^+$-MS/MS (S25.a.) and ESI$^-$-MS/MS (S25.b.) spectra of osirisyne B (**5**) with outlines of dissociation of the precursor ion.

S25.a.

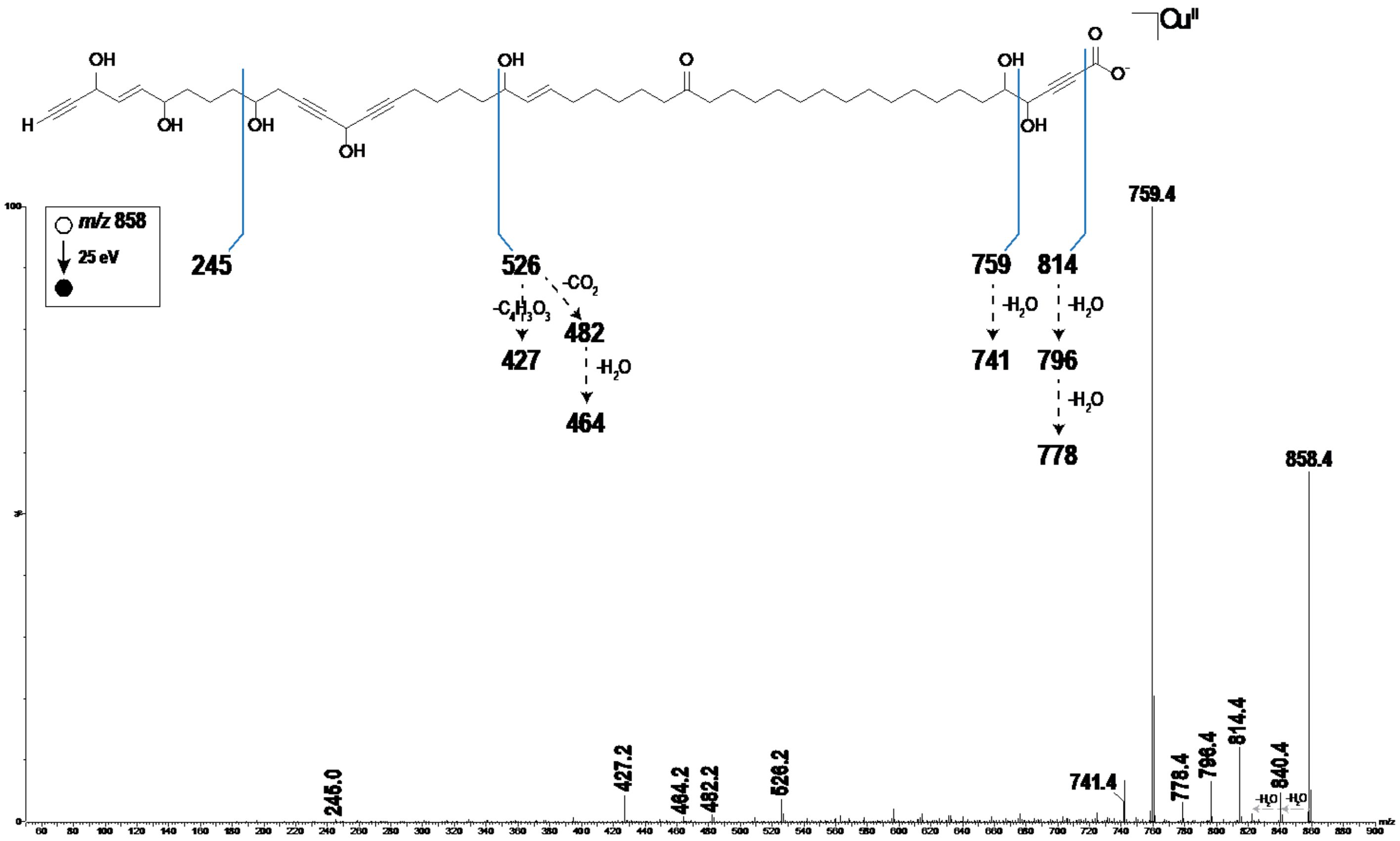

S25.b.

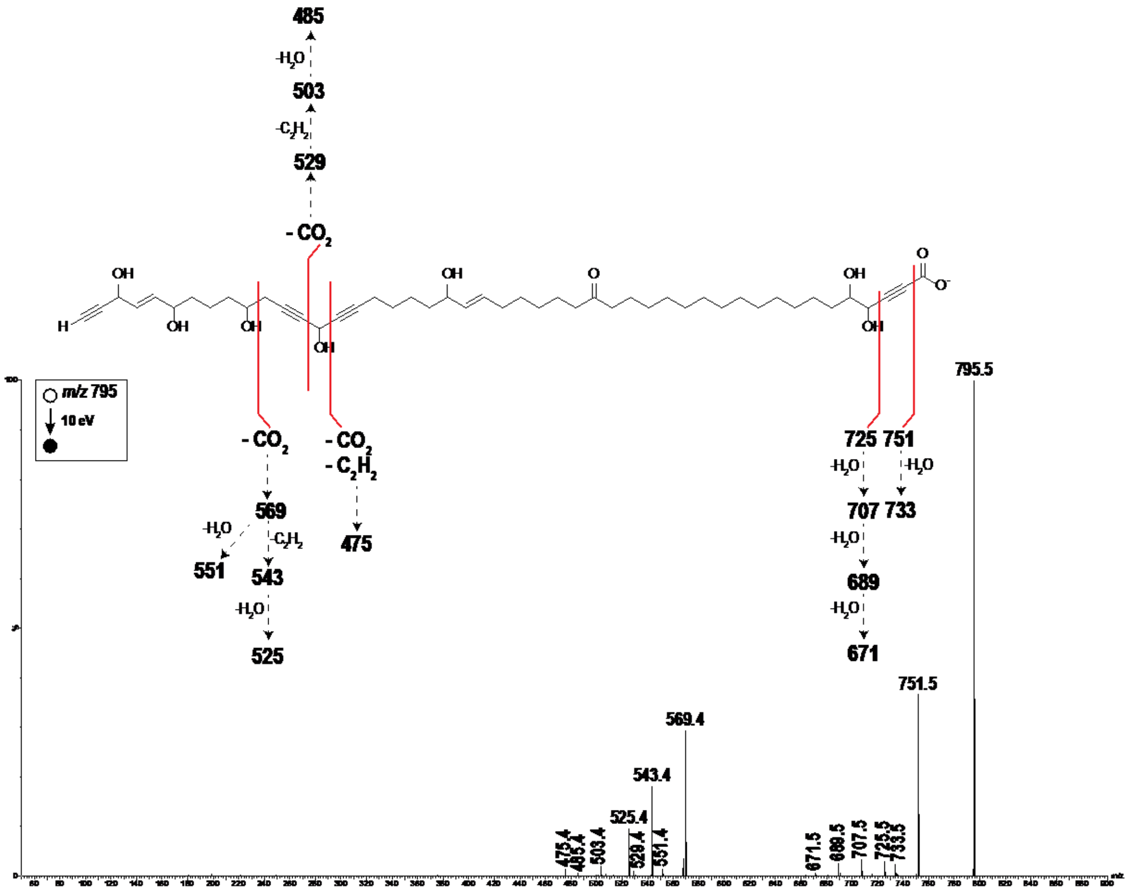

485
-H2O
503
-C2H2
529
- CO2
OH
OH
O
OH
O
O-
H
OH
OH
OH
OH
m/z 795
10 eV
795.5
- CO2
- CO2
- C2H2
725 751
-H2O
-H2O
707 733
569
-H2O
-C2H2
475
551
543
-H2O
689
-H2O
525
671
751.5
569.4
543.4
525.4
475.4
485.4
503.4
529.4
551.4
671.5
689.5
707.5
725.5
733.5

Figure S31: ^{1}H NMR (600 MHz, CD_3OD) spectrum for osirisyne B (**5**)

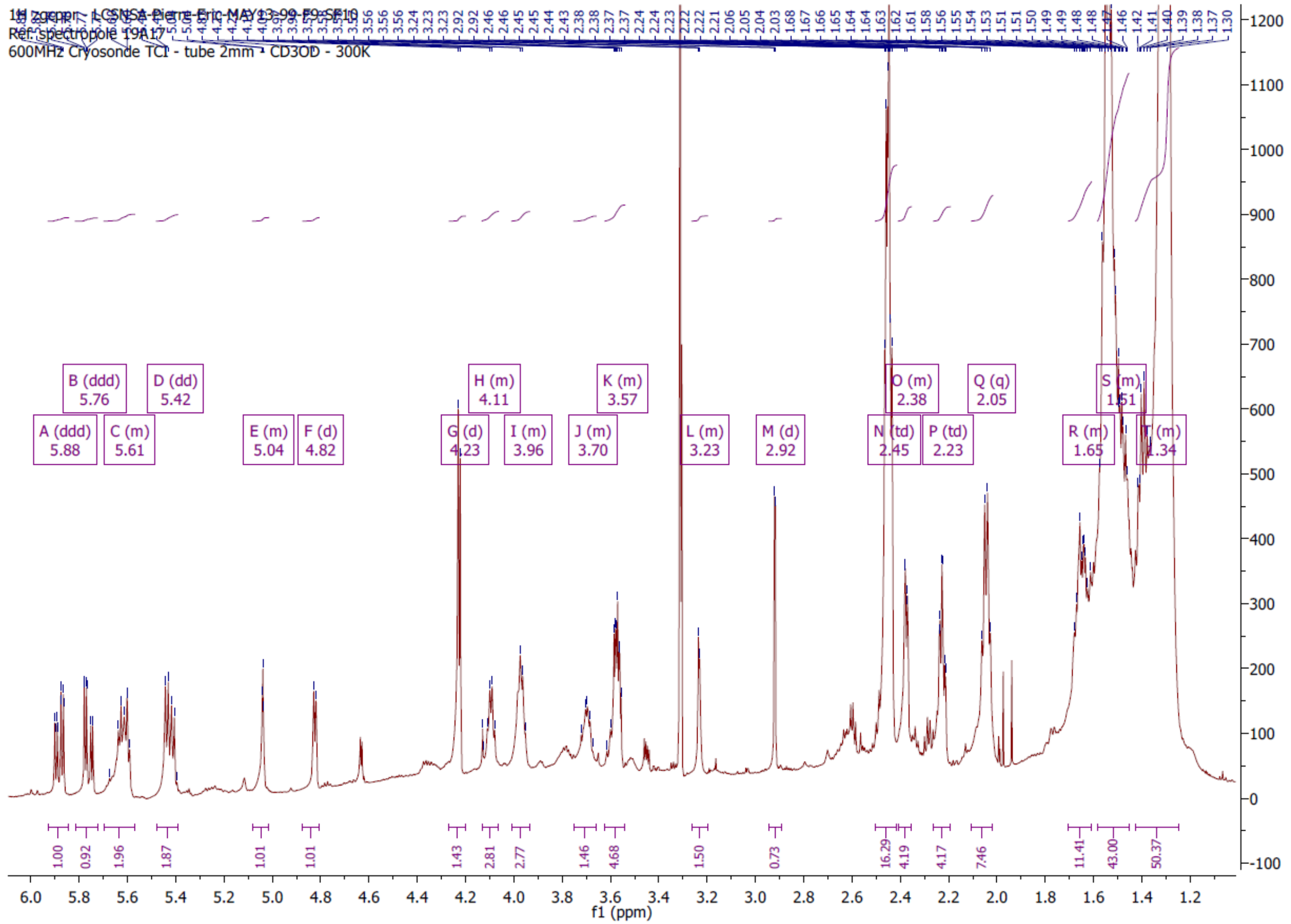

Figure S32: ^{13}C NMR (600 MHz, CD_3OD) spectrum for osirisyne B (**5**)

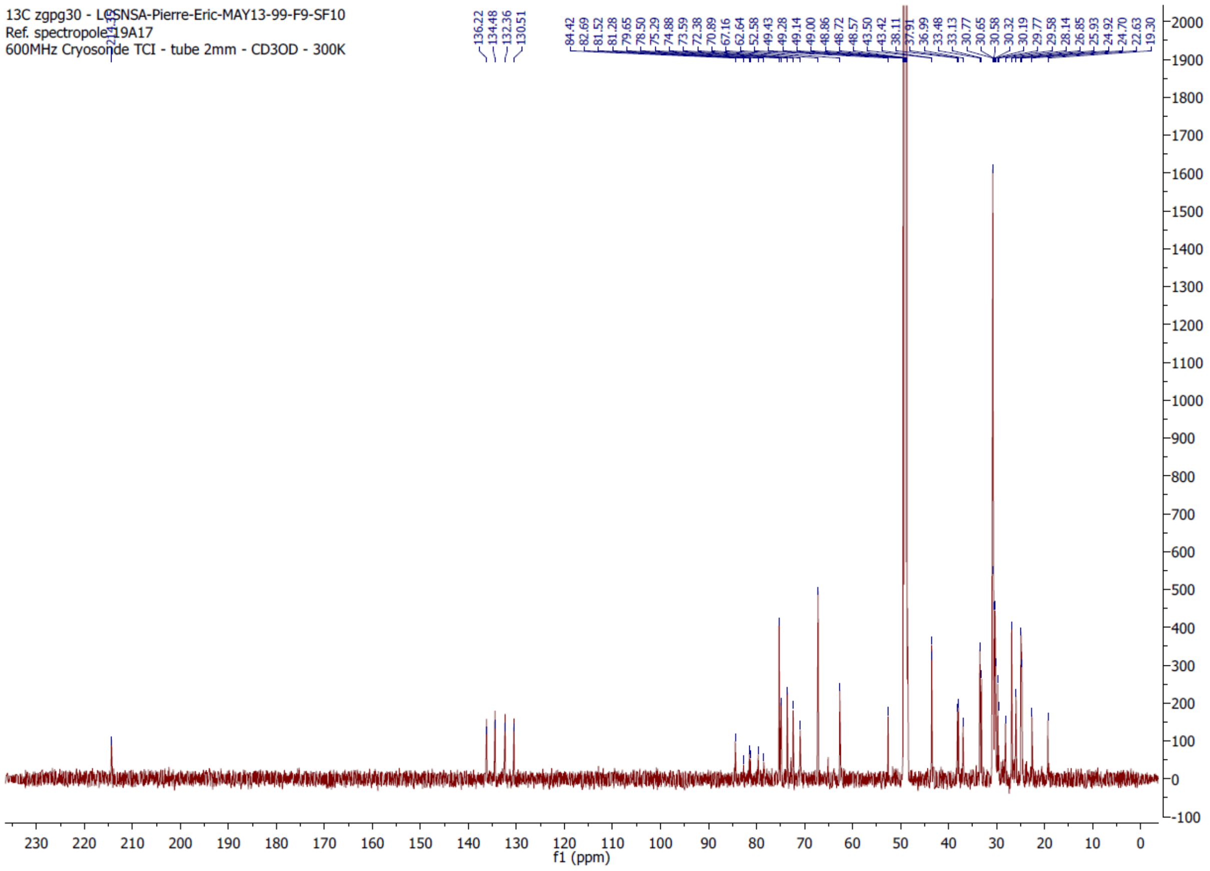

Deleted:

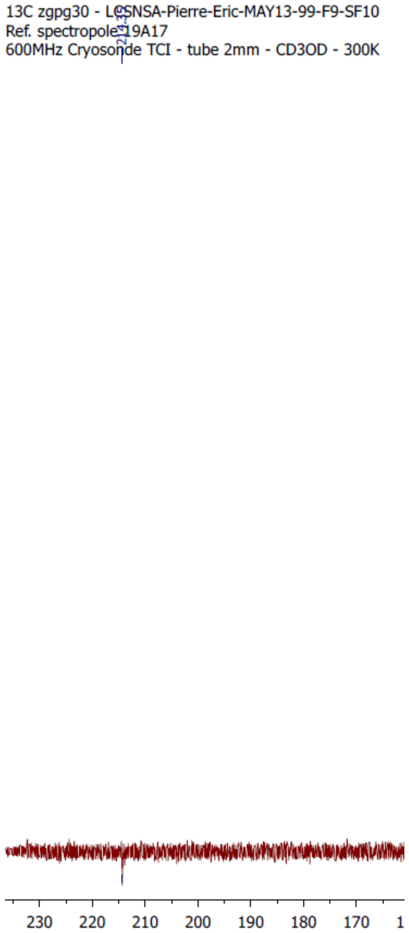

Figure S33: ^{1}H-^{1}H TOCSY NMR (600 MHz) spectrum for osirisyne B (**5**)

Formatted: Font: Bold

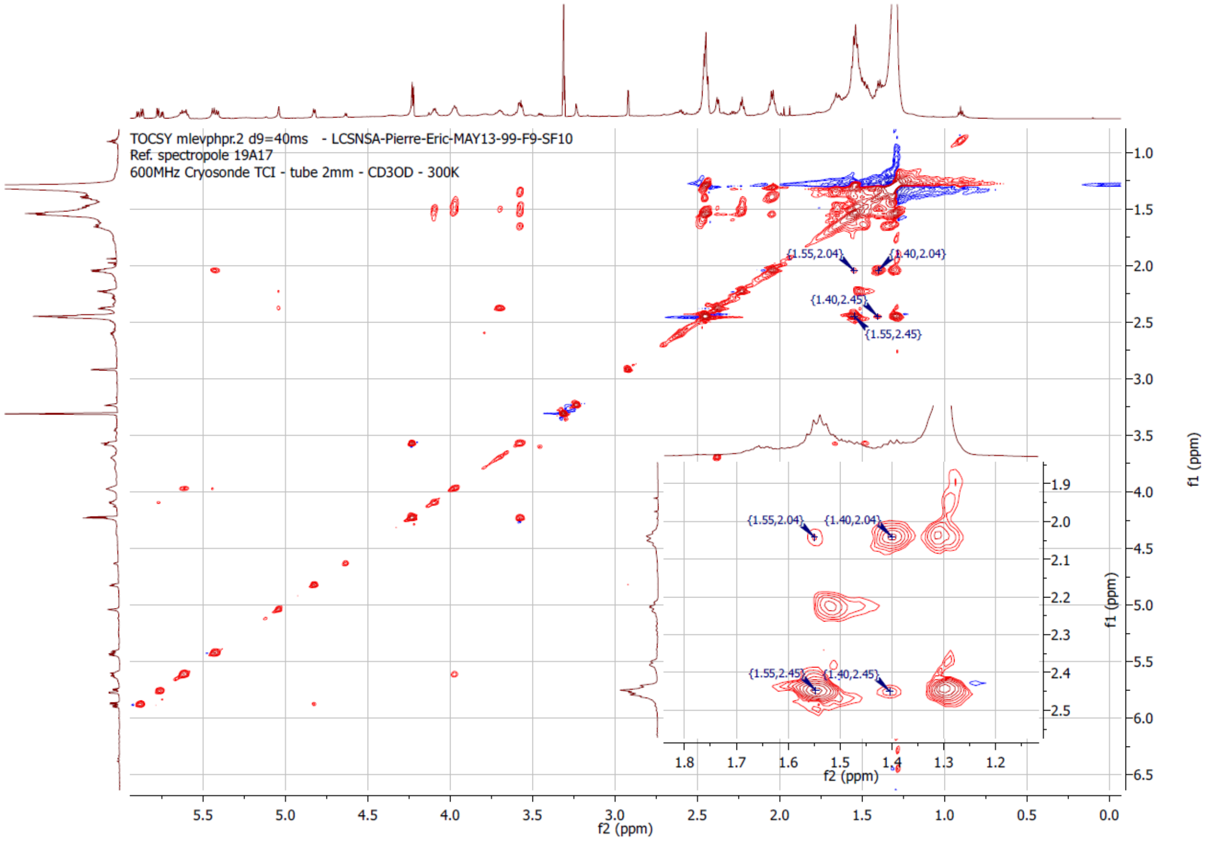

Figure S34: HRESIMS spectrum for osirisyne E (**6**)

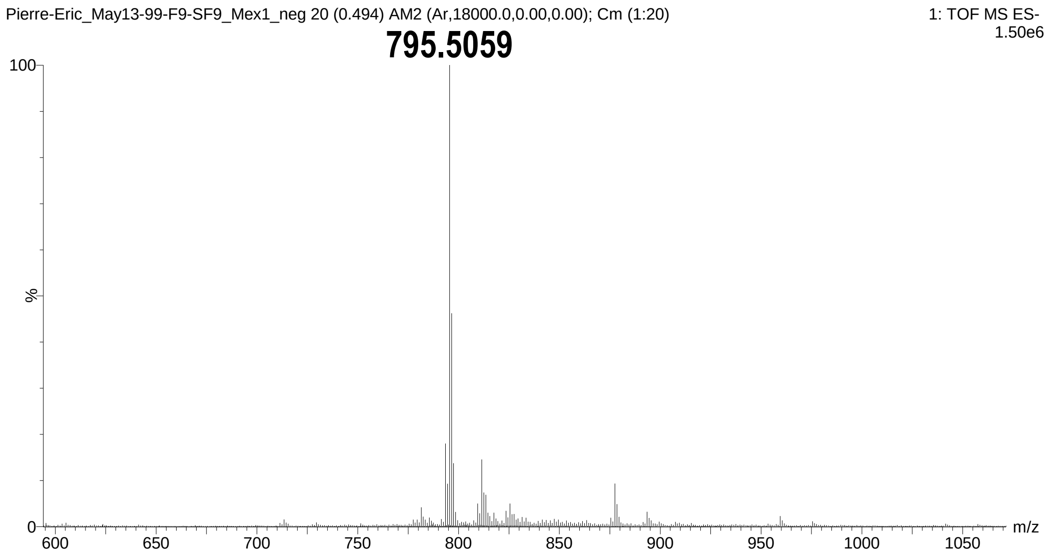

Figure S35*:* ESI$^+$-MS/MS (S28.a.) and ESI$^-$-MS/MS (S28.b.) spectra of osirisyne E (**6**) with outlines of dissociation of the precursor ion.

S28.a.

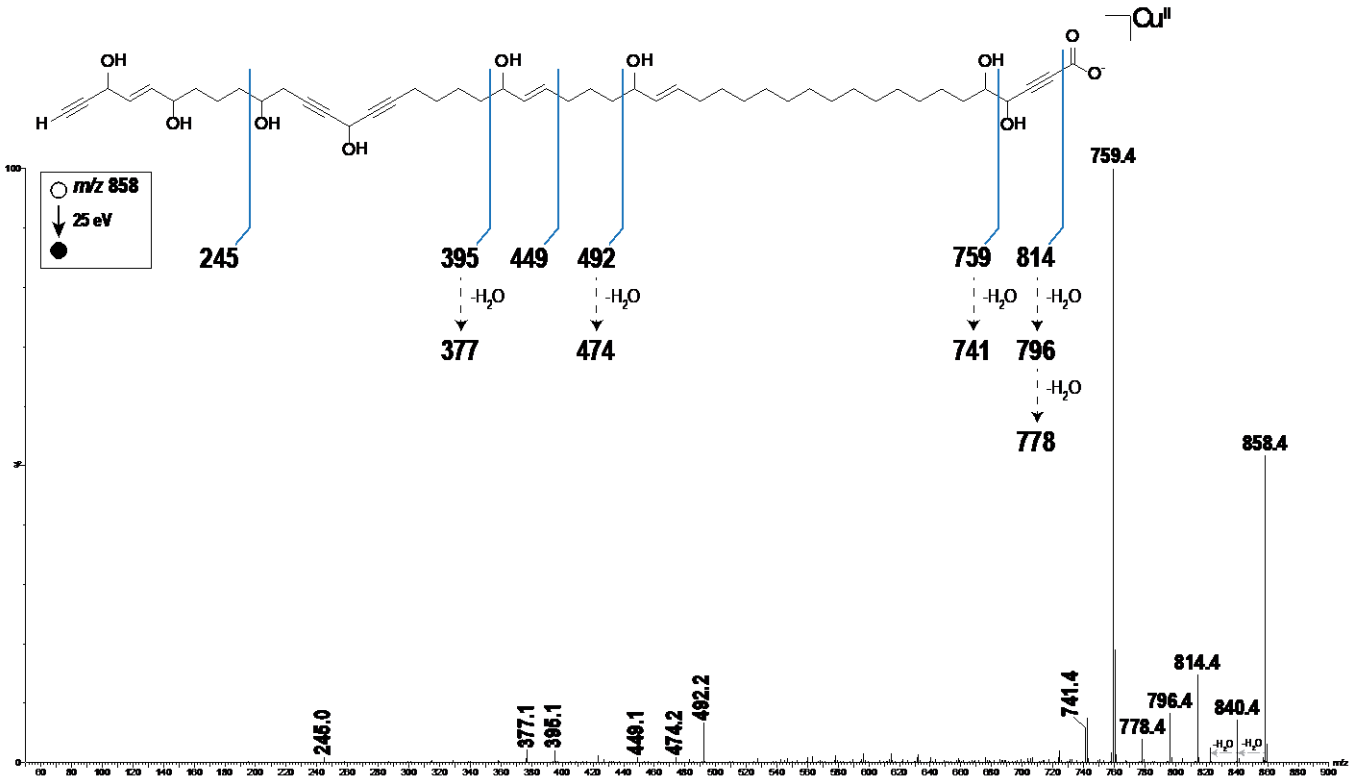

S28.b.

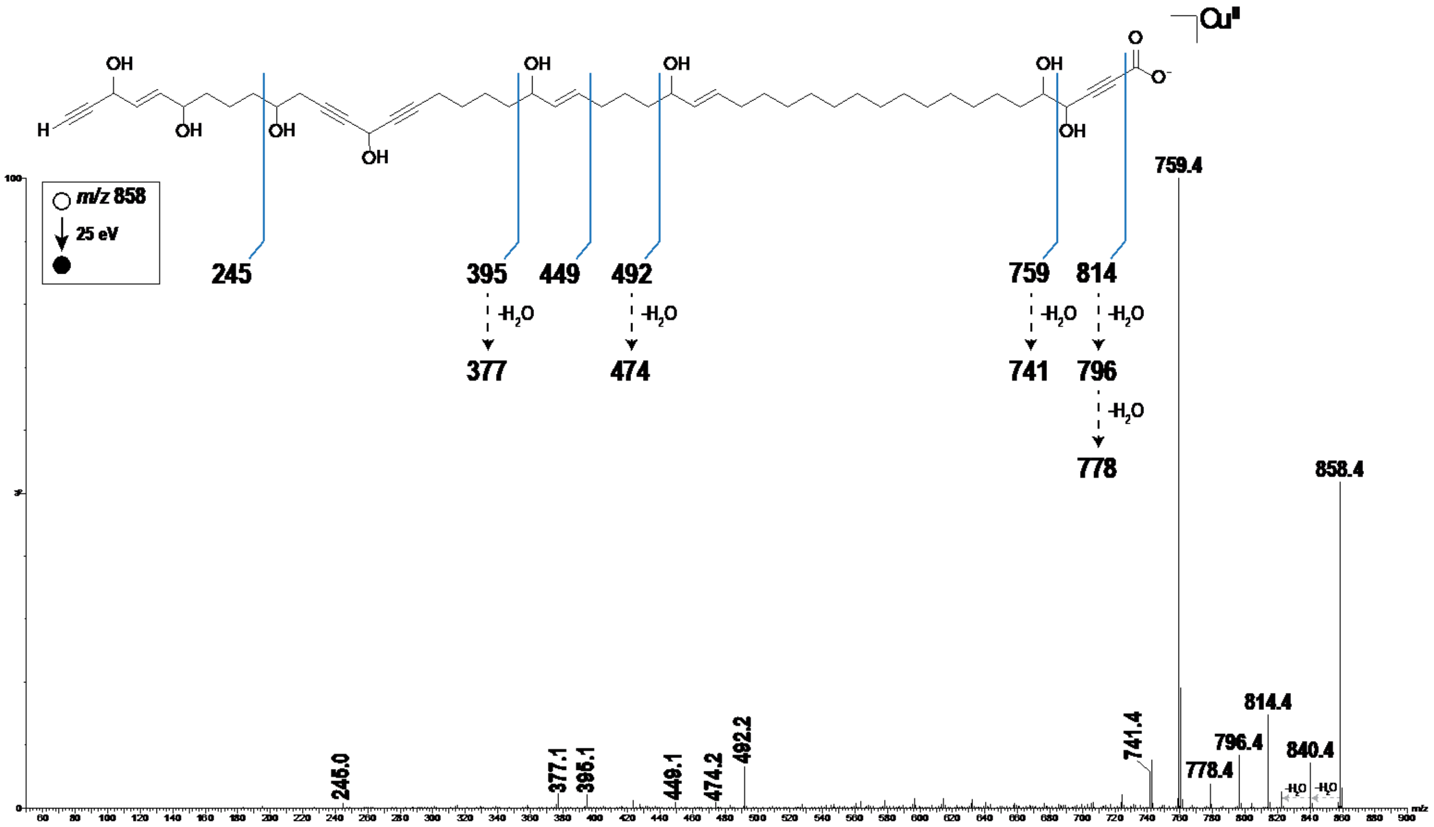

m/z 858
25 eV
245
395
449
492
759
814
-H2O
377
474
741
796
778
759.4
858.4
814.4
796.4
840.4
778.4
741.4
492.2
474.2
449.1
395.1
377.1
245.0
m/z

Figure S36: ^{1}H NMR (600 MHz, CD_3OD) spectrum for osirisyne E (**6**)

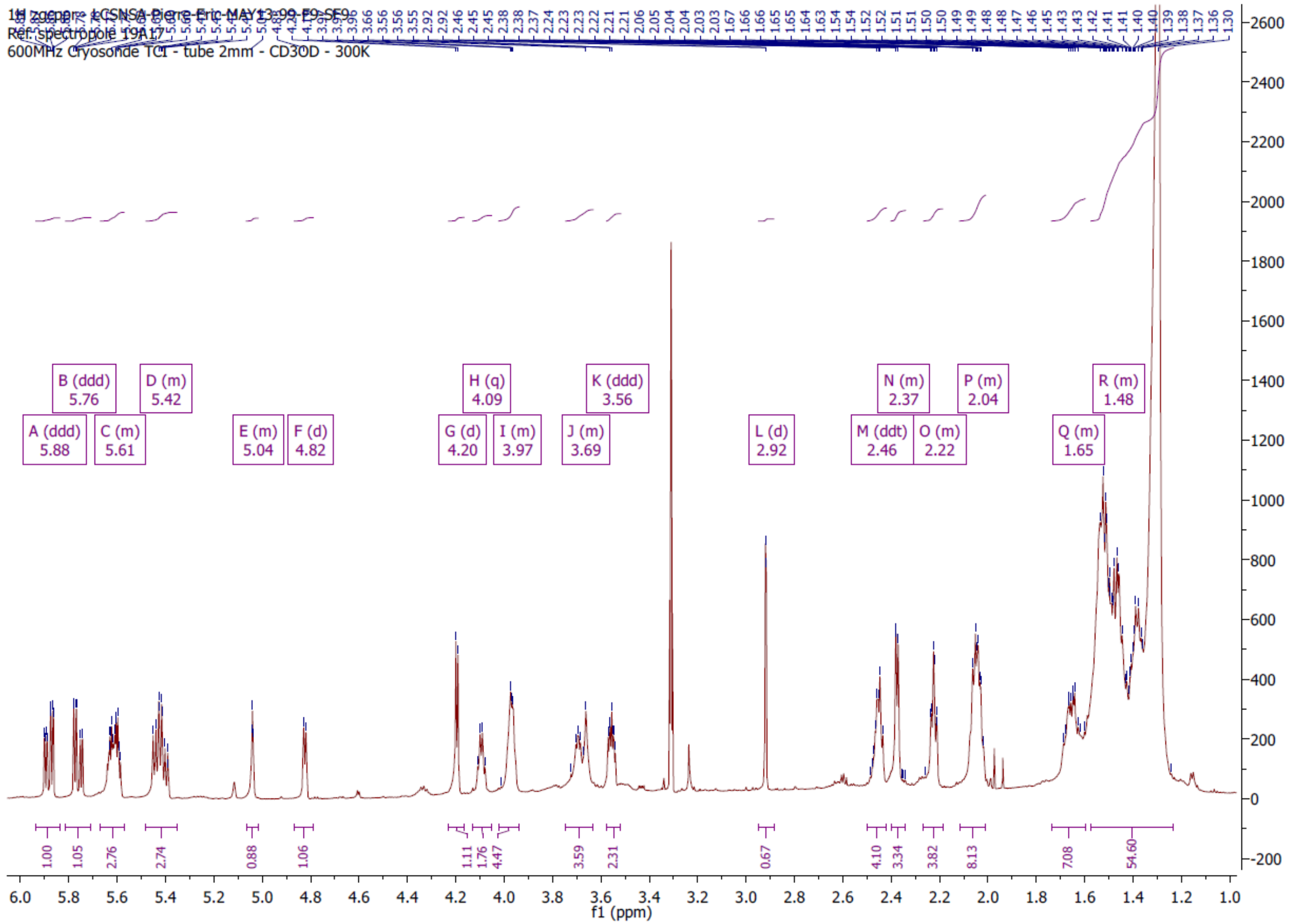

Figure S37: ^{13}C NMR (600 MHz, CD_3OD) spectrum for osirisyne E (**6**)

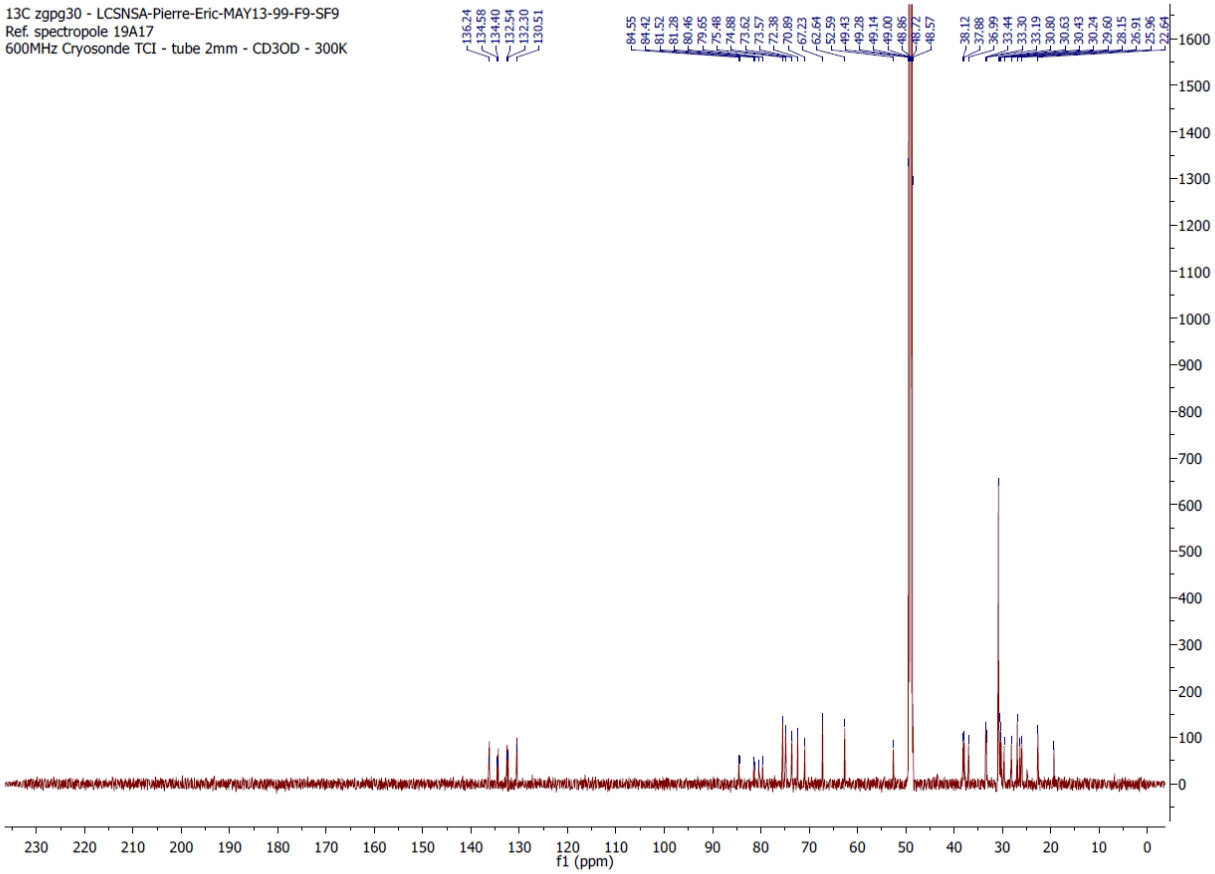

Deleted:

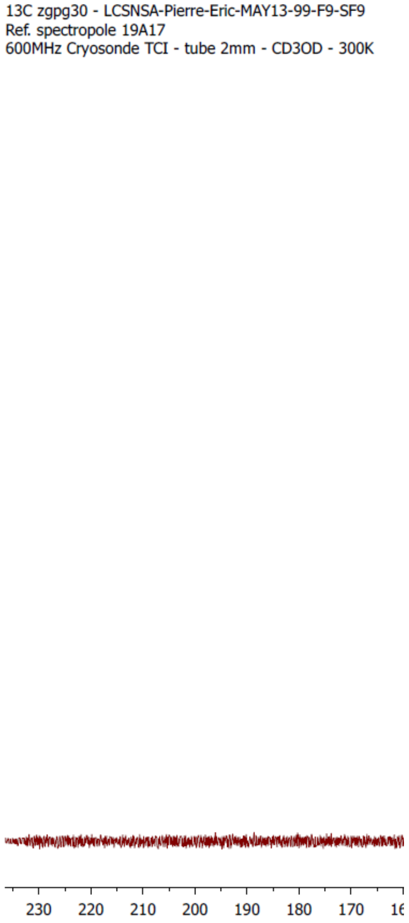

Supplement: Supplementary file 1 [file marinedrugs-18-00350-s001.pdf]
